# Dynamic modelling of the PI3K/MTOR signalling network uncovers biphasic dependence of mTORC1 activity on the mTORC2 subunit SIN1

**DOI:** 10.1371/journal.pcbi.1008513

**Published:** 2021-09-16

**Authors:** Milad Ghomlaghi, Guang Yang, Sung-Young Shin, David E. James, Lan K. Nguyen

**Affiliations:** 1 Department of Biochemistry and Molecular Biology, School of Biomedical Sciences, Monash University, Melbourne, Australia; 2 Biomedicine Discovery Institute, Monash University, Melbourne, Australia; 3 Charles Perkins Centre, School of Life and Environmental Sciences, The University of Sydney, Sydney, Australia; University of Southern California, UNITED STATES

## Abstract

The PI3K/MTOR signalling network regulates a broad array of critical cellular processes, including cell growth, metabolism and autophagy. The mechanistic target of rapamycin (MTOR) kinase functions as a core catalytic subunit in two physically and functionally distinct complexes mTORC1 and mTORC2, which also share other common components including MLST8 (also known as GβL) and DEPTOR. Despite intensive research, how mTORC1 and 2 assembly and activity are coordinated, and how they are functionally linked remain to be fully characterized. This is due in part to the complex network wiring, featuring multiple feedback loops and intricate post-translational modifications. Here, we integrate predictive network modelling, *in vitro* experiments and -omics data analysis to elucidate the emergent dynamic behaviour of the PI3K/MTOR network. We construct new mechanistic models that encapsulate critical mechanistic details, including mTORC1/2 coordination by MLST8 (de)ubiquitination and the Akt-to-mTORC2 positive feedback loop. Model simulations validated by experimental studies revealed a previously unknown biphasic, threshold-gated dependence of mTORC1 activity on the key mTORC2 subunit SIN1, which is robust against cell-to-cell variation in protein expression. In addition, our integrative analysis demonstrates that ubiquitination of MLST8, which is reversed by OTUD7B, is regulated by IRS1/2. Our results further support the essential role of MLST8 in enabling both mTORC1 and 2’s activity and suggest MLST8 as a viable therapeutic target in breast cancer. Overall, our study reports a new mechanistic model of PI3K/MTOR signalling incorporating MLST8-mediated mTORC1/2 formation and unveils a novel regulatory linkage between mTORC1 and mTORC2.

## Author summary

Signalling networks are the key information-processing machineries that underpin the ability of living cells to respond proportionately to extra- (and intra-) cellular cues. The PI3K/MTOR signalling network is one of the most important signalling networks in human cells that regulates cellular response to critical hormones such as insulin; yet our understanding of the network behaviour remains far from complete. Here, we employed a highly integrative approach that combines predictive mathematical modelling, biological experimentation, and data analysis to gain novel systems-level insights into PI3K/MTOR signalling. We constructed new mathematical models of this complex network incorporating important regulatory mechanisms. In contrary to commonly-held views that mTORC2 lies upstream and is a positive regulator of mTORC1, we found that their relationship is highly non-linear and dose dependent. This finding has major implications for anti-mTORC2 therapy as depending on the cellular contexts, inhibiting mTORC2 may either reduce or enhance mTORC1 activation, the latter could inadvertently dampen the effect of mTORC2 blockade. Furthermore, our results demonstrate that MLST8 is required for the assembly and activity of both MTOR complexes and suggest MLST8 is a viable therapeutic target in breast cancer.

## Introduction

The PI3K/MTOR signalling network plays an important role in the regulation of cell signal transduction and regulates a variety of key biological processes such as cell growth, metabolism and autophagy [1]. The mechanistic target of rapamycin (MTOR) is a Ser/Thr kinase that lies at the center of this complex network, where it serves as an indispensable catalytic subunit for two functionally distinct complexes termed MTOR complex 1 (mTORC1) and MTOR complex 2 (mTORC2). In addition to mTOR, mTORC1 and 2 share two common subunits, MTOR associated protein LST8 homolog (MLST8, also known as GβL) and DEP domain-containing mTOR-interacting protein (DEPTOR); whereas regulatory associated protein of MTOR complex 1 (raptor) and proline-rich Akt substrate of 40 kDa (PRAS40) are unique components of mTORC1 [2], and stress-activated map kinase-interacting protein 1 (SIN1) [3] and raptor independent companion of MTOR complex 2 (RICTOR) [4] are exclusive to mTORC2. Reflecting its importance in physiological regulation, the PI3K/MTOR network is frequently disrupted in human diseases, including cancer, metabolic and neurodegenerative disorders [2]. In cancer alone, more than 40 inhibitors directed at various components of the network have been developed or are under active development [5]. Given the clinical relevance of PI3K/MTOR signalling, it is important to understand the interconnectivities within this network and emergent network behaviors.

**Fig 1:**
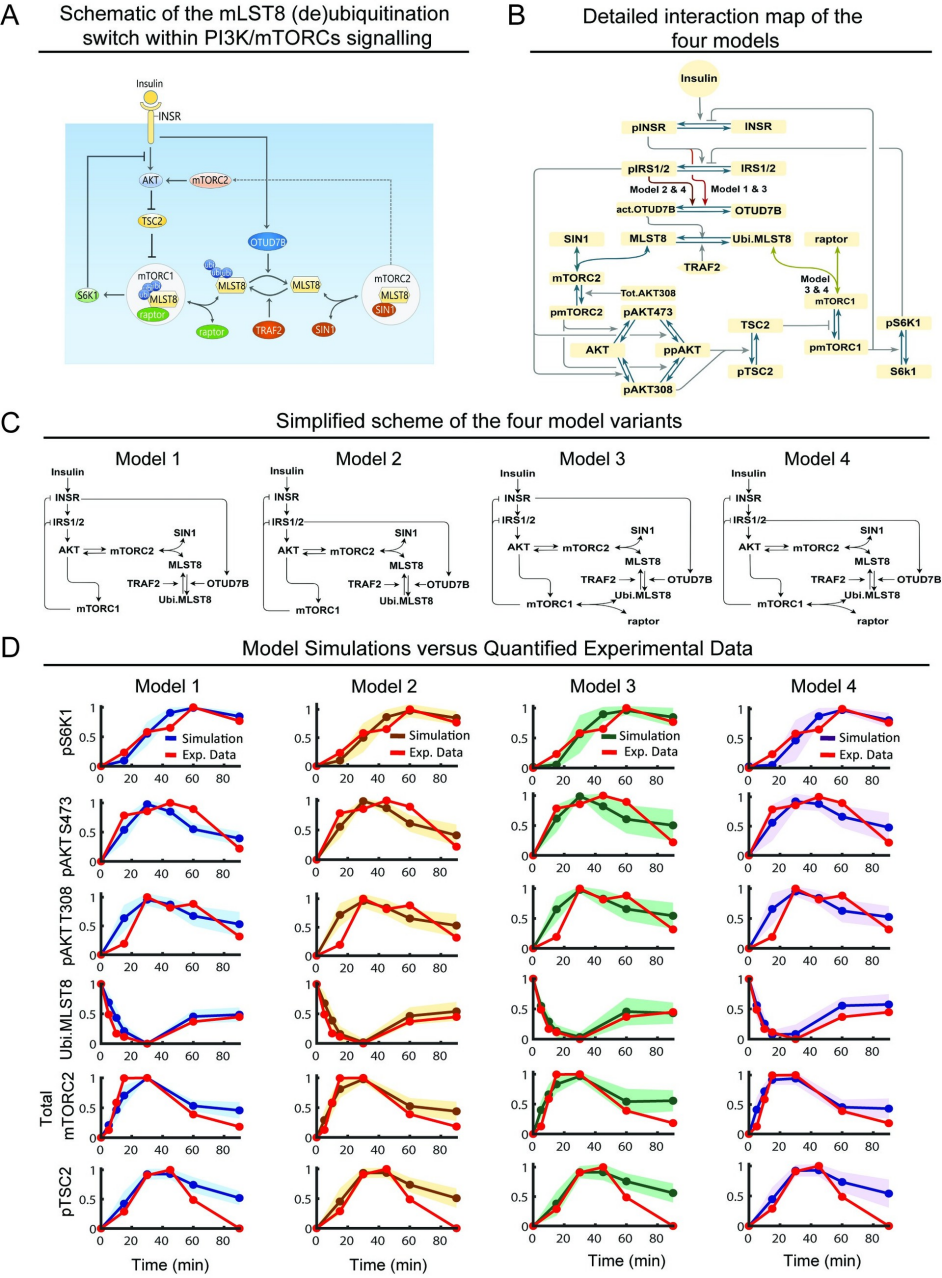
Construction and calibration of new mathematical models of the PI3K/MTOR signalling network incorporating the MLST8 (de)ubiquitination switch. (A) Schematic displaying a molecular switch mediated by MLST8 ubiquitination recently reported in [7], which dictates how MLST8 associates with mTORC1 or 2 through raptor and SIN1, respectively (ubi: ubiquitination). (B) Detailed reaction diagrams of the new PI3K/MTOR network models incorporating the MLST8 switch. Four model variants (models 1–4) with distinguishing interactions as described in the text are highlighted (ubi: ubiquitination, act: activated, Tot: total). (C) Simplified schematics of the different network structures captured by the four model variants. (D) Time-course model simulations using best-fitted parameter sets as compared to the quantified experimental data (red curves), presented for each of the four models. Experimental data represent the dynamic response of various network proteins following insulin stimulation (100nM) in MEF cells, reproduced from Western blot data representative of at least three biological replicates obtained from [7]. Per model, multiple best-fitted sets obtained from model calibration (n = 189, 198, 12, 13 for model 1, 2, 3, 4, respectively) were used for simulations: the lines represent the mean simulated curve and the shaded areas indicate standard deviation of all curves.

The PI3K/MTOR network is highly complicated and arguably one of the most extensively studied signalling pathways, yet its complexity continues to expand through new mechanistic discoveries. For example, in addition to known feedback mechanisms such as p70S6 kinase (S6K)-mediated negative feedback to PI3K/AKT via IRS1/2, we have identified a positive feedback loop between AKT and mTORC2, where AKT phosphorylates SIN1 to enhance mTORC2 activity [5,6]. More recently, a molecular switch involving MLST8 through its (de)ubiquitination modification was identified [7]. Mechanistically, the TNF receptor-associated factor 2 (TRAF2) E3 ubiquitin ligase promotes MLST8 ubiquitination on lysine 63 (K63), which disrupts its interaction with the unique mTORC2 component SIN1 [7]. By contrast, ubiquitinated MLST8 can be converted back to its de-ubiquitinated form by the OTU deubiquitinase 7B (OTUD7B) deubiquitinase. De-ubiquitinated MLST8 binds more favourably to SIN1, which facilitates mTORC2 assembly but at the same time reduces mTORC1 formation [7] (Fig 1A). These findings add extra layers of complexity and intricacy to the wiring of the PI3K/MTOR network, however its dynamic properties incorporating these new regulatory mechanisms have not been characterized.

Moreover, although the MLST8 ubiquitination-dependent switch seems to provide tight regulation of MTOR complex integrity, the functional role of MLST8 in each complex remains incompletely defined. While it is established that MLST8 is indispensable for mTORC2 activity [8,9], whether it is essential for mTORC1 function is unclear. For example, ablation of MLST8 does not affect mTORC1 activity as measured by the phosphorylation level of its major substrate S6K1[9–11]. On the other hand, MLST8 stabilizes the raptor-MTOR interaction and promotes mTORC1 activity [12], and upregulation of MLST8 enhances mTORC1/2 activities in colon carcinoma and prostate cancer [13].

Here we employed an integrated approach that combines predictive network modelling and biological experiments to analyze the emergent network-level behavior of PI3K/MTOR signalling conferred by the MLST8-mediated switch. We constructed alternative mechanistic models of the PI3K/MTOR network that explicitly encapsulate MLST8 (de)ubiquitination, ensuing mTORC1/2 coordination, and the AKT-mTORC2 positive feedback. The candidate models consider competing hypotheses of network interactions, reflecting different network structures. To differentiate among the models and arrive at the optimal one, we performed comparative model calibration and validation using time-resolved experimental data. Using the optimal model, we undertook simulation studies with an emphasis on the regulation of mTORC1/2 formation and activity, and characterized the governing factors through network perturbation analysis. Model predictions were validated experimentally using *in vitro* assays and interrogation of publicly available data.

Our integrative studies revealed a hitherto unknown biphasic dependence of mTORC1 activity on the key mTORC2 component SIN1, uncovering an emergent functional linkage between the two MTOR complexes. This non-linear dependence seems to be a robust feature among a broad array of cell types, as model simulations predict its existence in the face of marked cell-to-cell heterogeneity in protein expression. The SIN1-mTORC1 biphasic response may help explain context-specific biological observations in cells with low or high levels of SIN1. Furthermore, our results demonstrate that MLST8 is required for the assembly and activity of both MTOR complexes and suggest MLST8 is a viable therapeutic target in breast cancer. As cellular response specificity is encoded by the spatial and temporal dynamics of signalling networks and coordinated by all network components, this study highlights the need to undertake systems approaches to study complex perturbation-response relationship. To this end, the models developed here provide useful resources for future systems-based studies of PI3K/MTOR signalling.

## Results

### Construction of mechanistic PI3K/MTOR network models incorporating MLST8-mediated switch

To elucidate the functional role of the MLST8 (de)ubiquitination switch in coordinating mTORC1/2 formation and PI3K/MTOR network dynamics, we constructed new mathematical models of this network incorporating the switch regulation. A number of models have previously been developed for the PI3K/MTOR pathway [14]. For example, Dalle Pezze et al. (2012) presented a model using ordinary differential equations (ODEs) to investigate potential regulators of mTORC2 [15]. Based on observations that amino acids can also activate mTORC2 in addition to mTORC1 [16], an integrated modelling-experimental approach was employed to identify novel downstream targets of amino acids within the MTOR pathway [17]. Other models focused on insulin resistance and aimed to explain the PI3K/MTOR network dynamics following insulin stimulation in healthy and diabetic cells [18–21]. We previously constructed mechanistic models that revealed complex emergent network dynamics conferred by DEPTOR, an endogenous inhibitor of both mTORC1 and 2 [22]. However, none of the published models consider the coordination of mTORC1/2 formation by the MLST8 (de)ubiquitination switch [7]. The models developed in this study are the first to explicitly incorporate this switch, in addition to known regulatory mechanisms. Below we describe the key model assumptions and underpinning experimental observations.

### MLST8-mediated switch regulates MTOR complex formation

Although MLST8 was identified as a shared component of mTORC1 and 2 more than a decade ago [12,23], only recently the (de)ubiquitination of MLST8 was found to regulate its binding to mTORC1 and 2 [7]. Depicted in Fig 1A, MLST8 is ubiquitinated by the E3 ligase TRAF2 on its WD7 kinase domain, which is also the binding site for SIN1. Consequently, MLST8 ubiquitination prevents SIN1-MLST8 association and decreases formation of mTORC2. However, as raptor binds MLST8 via its WD6 domain, MLST8 ubiquitination does not affect MLST8-raptor binding [7]. Moreover, ubiquitination-mediated reduction of SIN1-MLST8 association frees MLST8 to be available for raptor binding, thereby enhancing mTORC1 formation [7]. The ubiquitination of MLST8 is reversed by the deubiquitinating enzyme OTUD7B, which catalyzes MLST8 deubiquitination [7]. By doing so, OTUD7B enhances MLST8’s binding affinity for SIN1 and thus mTORC2 formation (Fig 1A). Importantly, insulin acts as a triggering input for OTUD7B as insulin stimulation promotes activation of OTUD7B [7]. Collectively, MLST8 (de)ubiquitination functions as a molecular switch where ubiquitination promotes mTORC1 formation while simultaneously blocking mTORC2 formation, and deubiquitination does the opposite. However, how the MLST8-induced switch interplays with other regulatory mechanisms within the PI3K/MTOR network to orchestrate network behaviour is not clear. These mechanistic details are captured in our new models, indicated in the model reaction scheme in Fig 1B.

### Other key signalling events and feedback loops

The model schematic in Fig 1B further includes key signalling and feedback events induced by insulin stimulation. Briefly, insulin binds to the insulin receptor (INSR), triggering INSR dimerization, autophosphorylation and activation [24]. Activated INSR recruits and phosphorylates insulin receptor substrates (IRS1/2) that leads to PI3K recruitment and activation [24]. Activated PI3K in turn phosphorylates phosphatidylinositol (3,4,5)-bisphosphate (PIP2) and generates phosphatidylinositol (3,4,5)-trisphosphate (PIP3), which recruits the kinase 3-phosphoinositide-dependent protein kinase 1 (PDK1) to the plasma membrane to phosphorylate AKT at Threonine 308 (pAKT T308). To keep the model simplified without compromising dynamic accuracy, we lumped the IRS1/2 → PI3K → PIP3 → PDK1 → pAKT T308 cascade into a single step, IRS1/2 → pAKT T308 (Fig 1B).

As an AGC family kinase, AKT requires dual phosphorylation to become fully activated [25]. To this end, mTORC2 serves as a second AKT kinase and phosphorylates it at Serine 473 (pAKT S473). We assume that double phosphorylated AKT (ppAKT) can be achieved independently through either pAKT T308 or pAKT S473 first (Fig 1B) [26]. Moreover, as pAKT S473 alone possesses relatively much weaker kinase activity compared to pAKT T308 and ppAKT [26], we assumed that phosphorylation of the downstream substrate TSC2 is primarily catalyzed by the latter. This acts to inhibit TSC2 and releases its repression on mTORC1 activity. Activated mTORC1 phosphorylates S6K1, which in turn phosphorylates IRS1/2 on the inhibitory sites S636/S639, forming a well-established negative feedback within the PI3K/MTOR pathway that downregulates the input signal [27]. In addition, there is another negative feedback from mTORC1 to INSR via growth factor receptor bound protein 10 (Grb10) [28,29] (Fig 1B).

The study by Liu et al. [30] indicates that S6K1phosphorylates SIN1 at Threonine 86 and 398 and impairs mTORC2 function. However, our previous studies instead demonstrate that AKT, rather than S6K1, actually phosphorylates SIN1 at Threonine 86 (T86), which acts to enhance mTORC2 kinase activity. This generates a positive feedback loop between mTORC2 and AKT [5], which was incorporated into the model.

### Construction of multiple network model variants

In addition to the more established signalling events above, there are gaps in our network structure understanding. First, although insulin stimulation was shown to promote OTUD7B activation, it is unclear if this is mediated at the level of the insulin receptor or downstream. Therefore, we constructed two different model variants to examine alternative scenarios: *(i)* in model 1, OTUD7B is regulated directly by INSR and thus is not influenced by IRS1/2 and the S6K1-IRS1/2 negative feedback loop; and (*ii*) in model 2, OTUD7B is regulated by IRS1/2 and therefore under the control of two negative feedbacks (Fig 1C, left panels). Second, while MLST8 is critical for mTORC2 kinase activity as previously discussed, the evidence regarding whether it is required for mTORC1’s functional activity are conflicting [9,10,12,13]. This led us to construct two additional models in order to investigate the role of MLST8 in mTORC1 regulation. Specifically, in models 1–2, we assumed that MLST8 is not required for mTORC1’s function. In contrast, in models 3–4, MLST8 binds to raptor and forms mTORC1, thus MLST8 is required for mTORC1 formation and activity (Fig 1C, right panels).

In summary, we constructed four model variants differing in specific details pertaining to the regulation of OTUD7B and the role of MLST8 in mTORC1 regulation, allowing us to examine competing hypotheses. The new models were formulated using ODEs that represent biochemical interactions as a series of ordinary differential equations based on established kinetic laws [31]. Solving these equations enables us to evaluate temporal evolution in the concentration (i.e. states) of the network proteins. The rates of protein-protein interactions (e.g. association and dissociation reactions) were described by mass-action kinetics, and the rates of enzyme-catalyzed reactions (e.g. (de)phosphorylation and (de)ubiquitination) were given by Michaelis-Menten kinetics. Detailed description of the models, including ODE equations, reaction rates and model parameters are given in S1 and S2 Tables and S1 File.

### Model simulation predicts OTUD7B is regulated by IRS1/2

To confer specificity and predictive power to our models, we performed model calibration (i.e. parameter estimation) using insulin-stimulated time-course experimental data obtained from mouse embryonic fibroblast (MEF) cells previously published in [7], which was the primary cell model used for the characterization of the MLST8-mediated switch. The representative data were quantified using the software ImageJ [32] (see S1 Fig). Parameter estimation was carried out using an optimization procedure based on a genetic algorithm implemented in MATLAB (Materials and Methods). To avoid possible biases associated with using a single best-fitted parameter set, for each model we repeated the parameter estimation process 500 times to obtain multiple optimal parameter sets that best fitted the data, and utilized these sets collectively for subsequent simulation and analysis (see Materials and Methods).

**Fig 2:**
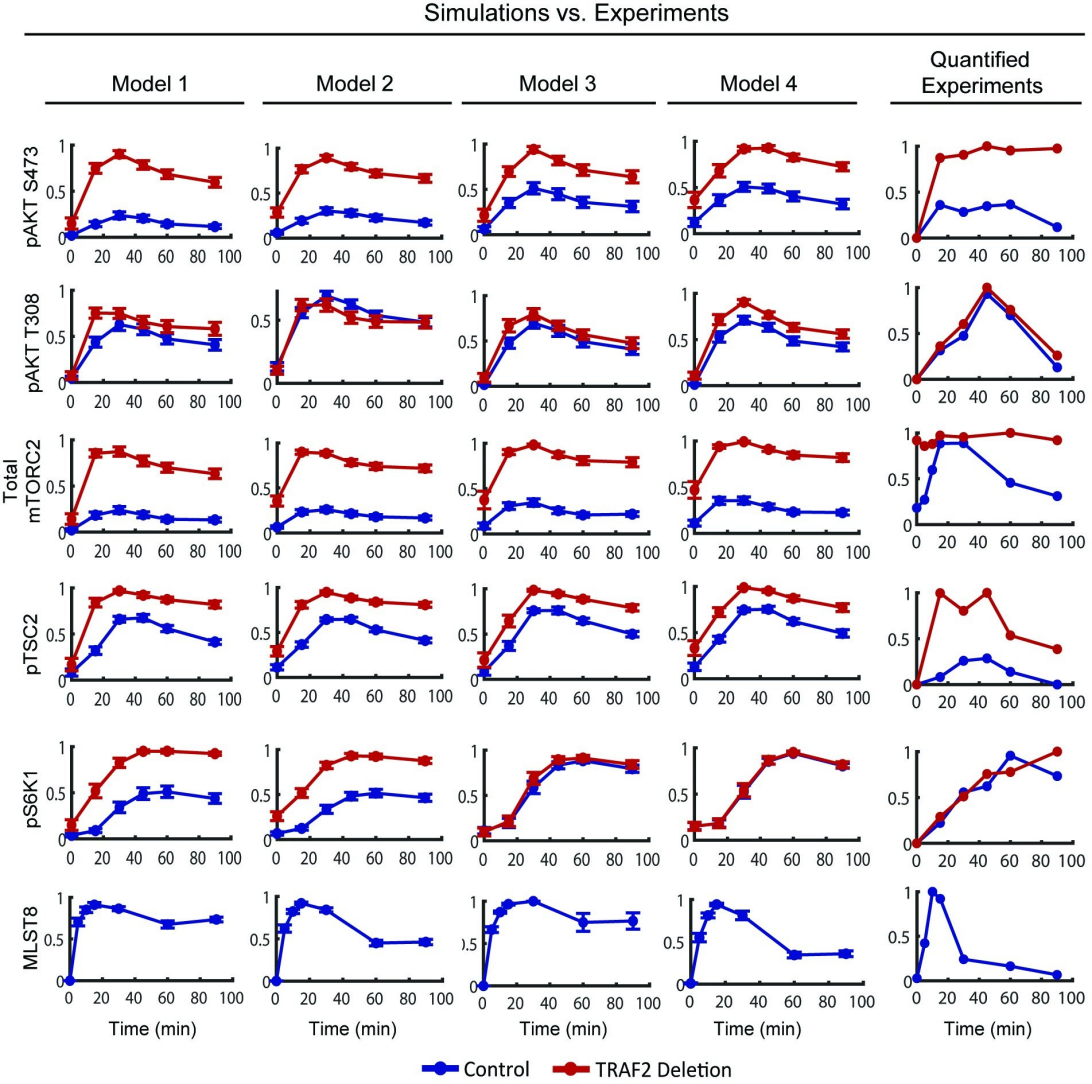
Independent validation of the four models. Simulations of the dynamic response of various network components to insulin stimulation in control (WT MEF cells, blue lines) condition and when TRAF2 is deleted (*TRAF2*^-/-^ MEF cells, red lines), in comparison to the corresponding experimental data (right panels), taken and quantified from [7]. The simulation data are presented as mean ± S.E.M. Experimental data points were reproduced from representative data of at least three biological replicates obtained from [7]. These validation analysis indicates models 1–3 could not recapitulate all data, and model 4 represents the best-fit model (see text for details).

Simulation results of the four models using the corresponding optimized parameter sets demonstrate all of the models recapitulate the experimental data well (Fig 1D). Next, to further assess the accuracy of these models, we compared model simulations with experimental data that was not used in the calibration process. To this end, we utilized insulin-stimulated time-course data from *TRAF2*^*-/-*^ MEF cells where the E3 ligase TRAF2 is silenced [7] (Fig 2, right panels). In addition, the temporal dynamics of MLST8 following insulin stimulation in wild-type (WT) MEF cells was not included in the calibration process and therefore was also used for model validation.

To mimic TRAF2 silencing, we reduced the concentration of TRAF2 by 90% of its WT level in the models. Comparing model simulations for *TRAF2*^*-/-*^ against the WT condition showed the models can qualitatively reproduce the temporal dynamics of phosphorylated AKT, TSC2 and mTORC2 (Fig 2). However, models 1 and 2 could not recapitulate the experimentally observed dynamics of phosphorylated S6K1, which was essentially not affected by TRAF2 knockout (Fig 2), whereas models 3 and 4 correctly captured this data. In addition, comparing simulated dynamics of unubiquitinated MLST8 with the experimental data showed models 1 and 3 failed to capture the strong overshoot pattern and low level of MLST8 at the late time-points (Fig 2, bottom panels). In contrast, these features were reproduced by models 2 and 4, which differ from models 1 and 3 in how OTUD7B is regulated (Fig 1C). While in models 1 and 3 only one negative feedback loop (from mTORC1 to INSR) coordinates OTUD7B activity; in models 2 and 4, OTUD7B is downstream of pIRS1/2 and thus regulated by two negative feedback loops (from mTORC1 to INSR, and S6K1 to IRS1/2). Given MLST8 is a direct substrate of OTUD7B, the regulation of OTUD7B by multiple negative feedback mechanisms in models 2 and 4 likely enables these models to better reflect the strong overshoot in MLST8 dynamics. Collectively, these computational analyses suggest OTUD7B is regulated through IRS1/2 rather than INSR directly, and indicate model 4 as the most probable model based on its superior ability to reproduce multiple sets of experimental data.

### Experimental validation of OTUD7B regulation by IRS1/2

**Fig 3:**
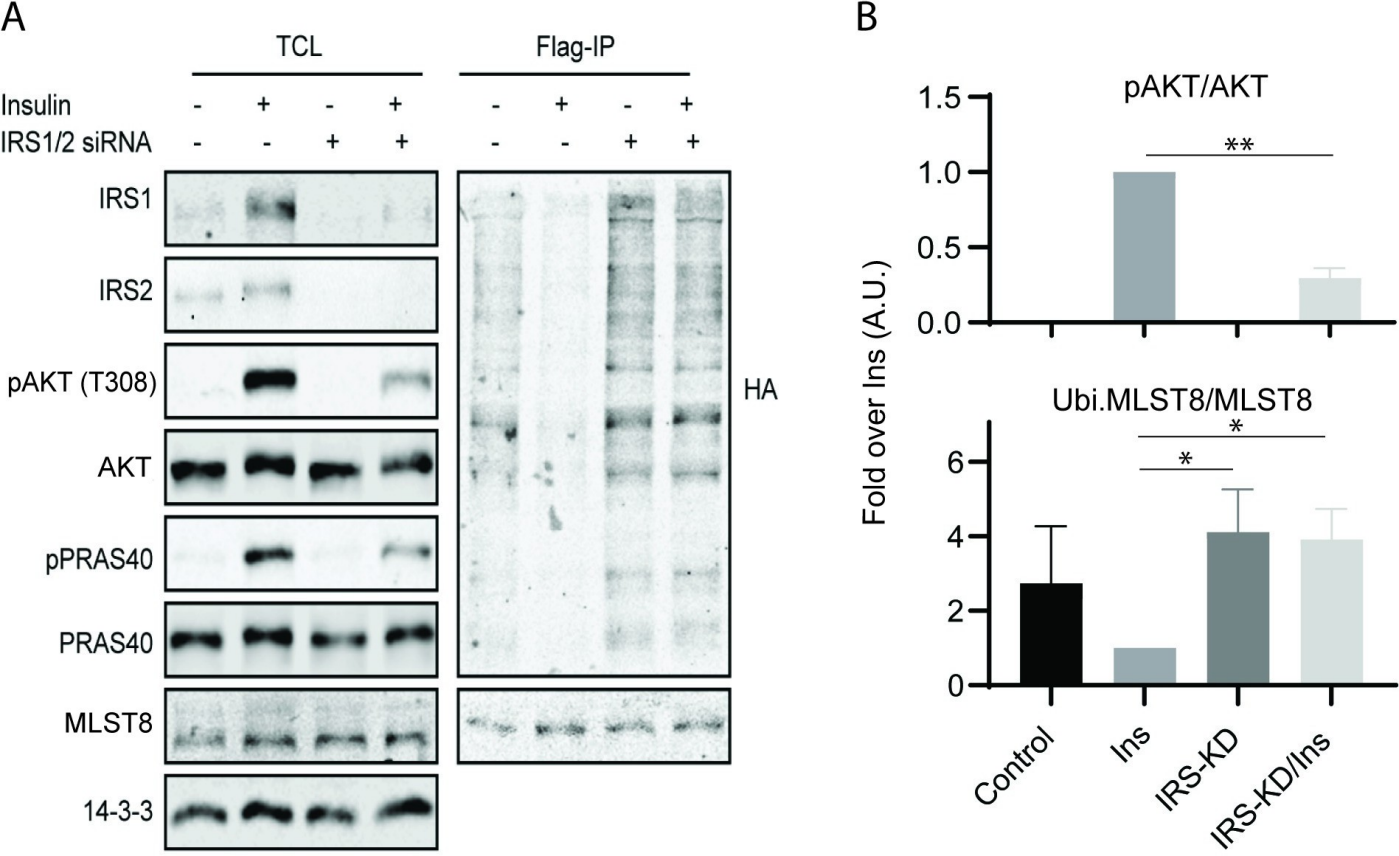
Experimental validation of IRS1/2-mediated regulation of MLST8 ubiquitination. (A) Flag-MLST8 immunoprecipitates from IRS1/2 knockdown C2C12 cells after insulin stimulation were analyzed by Western blot. (B) Graph shows mean ± S.E.M. of quantitative analyses of the Western blots in Fig 3A normalized for the total proteins (A.U., arbitrary units. * P<0.05, **P<0.01, two-tailed student’s t test, n = 3 biological replicates).

To experimentally validate the involvement of IRS1/2 in the regulation of OTUD7B, we evaluated the ubiquitination level of MLST8 upon siRNA-mediated depletion of IRS1 and IRS2. As shown in Fig 3, in C2C12 myoblast cells with control siRNA, the level of ubiquitinated MLST8 measured by HA-ubiquitin blotting was markedly reduced after acute insulin stimulation, which is consistent with previously reported studies [7] and confirms the role of insulin as a stimulating signal of OTUD7B’s deubiquitinase activity. Importantly, IRS1/2 depletion profoundly and significantly increased the level of ubiquitinated MLST8 in the presence of insulin stimulation (Fig 3B), suggesting IRS1/2 is an upstream regulator of OTUD7B activity. Moreover, both the phosphorylated levels of AKT and its major substrate PRAS40 were potently inhibited by siRNA-mediated IRS1/2 depletion, indicating the siRNA not only successfully downregulated IRS1/2 expression but also its functional activity. These data validate our model prediction and further support the validity of model 4.

### MLST8 is required for mTORC1 and mTORC2 activities

To understand why model 4 fitted experimental data best, we examined the time-course data more closely. While TRAF2 silencing led to a marked upregulation of both pAKT S473 and pTSC2 (Fig 3 for insulin stimulation, and S2A and S2B Fig for EGF stimulation), this did not really affect the level of phosphorylated S6K1 (Figs 3 and S2C). This seems counterintuitive since increased pTSC2 will release more mTORC1, resulting in increased mTORC1 activity and phosphorylation of S6K1, a direct mTORC1 substrate (see S3A Fig for a visual illustration of this reasoning). This raises the questions as to why pS6K1 was buffered from TRAF2 silencing, and which mechanism underpins such buffering?

We hypothesize that the stable pS6K1 level in response to TRAF2 knockout is due to a compensatory upregulation of mTORC1 activity. Model 4 assumes that MLST8 is required for mTORC1 function. Thus, while there are increased levels of mTORC2 and phosphorylated AKT and TSC2 in *TRAF2*^*-/-*^ cells, which together activate mTORC1 more strongly, the abundance of mTORC1 is reduced due to a loss of ubiquitinated MLST8. Overall, this leads to a balance in mTORC1’s total kinase activity potential (defined by the product of mTORC1 abundance and activity potential per mTORC1 molecule, as described in the Material and Methods), and so no changes in phosphorylated S6K1 (S3B Fig). This balance breaks down in models 1–2, which assume MLST8 is not required for mTORC1 function, resulting in a significant change in pS6K1 when TRAF2 is silenced.

Taken together, through rational construction of a series of mechanistic models and contrasting model simulations with experimental measurements, we arrived at model 4 that best reconciles multiple sets of experimental data in WT and *TRAF2*^*-/-*^ MEF cells. This model will be used for subsequent analyses.

### Modelling predicts biphasic mTORC1 activation dependency on mTORC2 subunit SIN1

Given the key role of MLST8 in coordinating the assembly and activity of mTORC1/2, perturbation of the MLST8-mediated switch is likely to disrupt mTORC1/2 signalling but how this occurs is not understood. To address this, we performed *in silico* sensitivity analysis where the concentration of the switch-related proteins (MLST8, TRAF2, OTUD7B, SIN1 and raptor) were systematically perturbed over a wide range (from 100-fold down to 1000-fold up) of their nominal values, and the impact on mTORC1 and 2 signalling was quantified using the response of pS6K1 and pAKT S473 at steady state, respectively.

**Fig 4:**
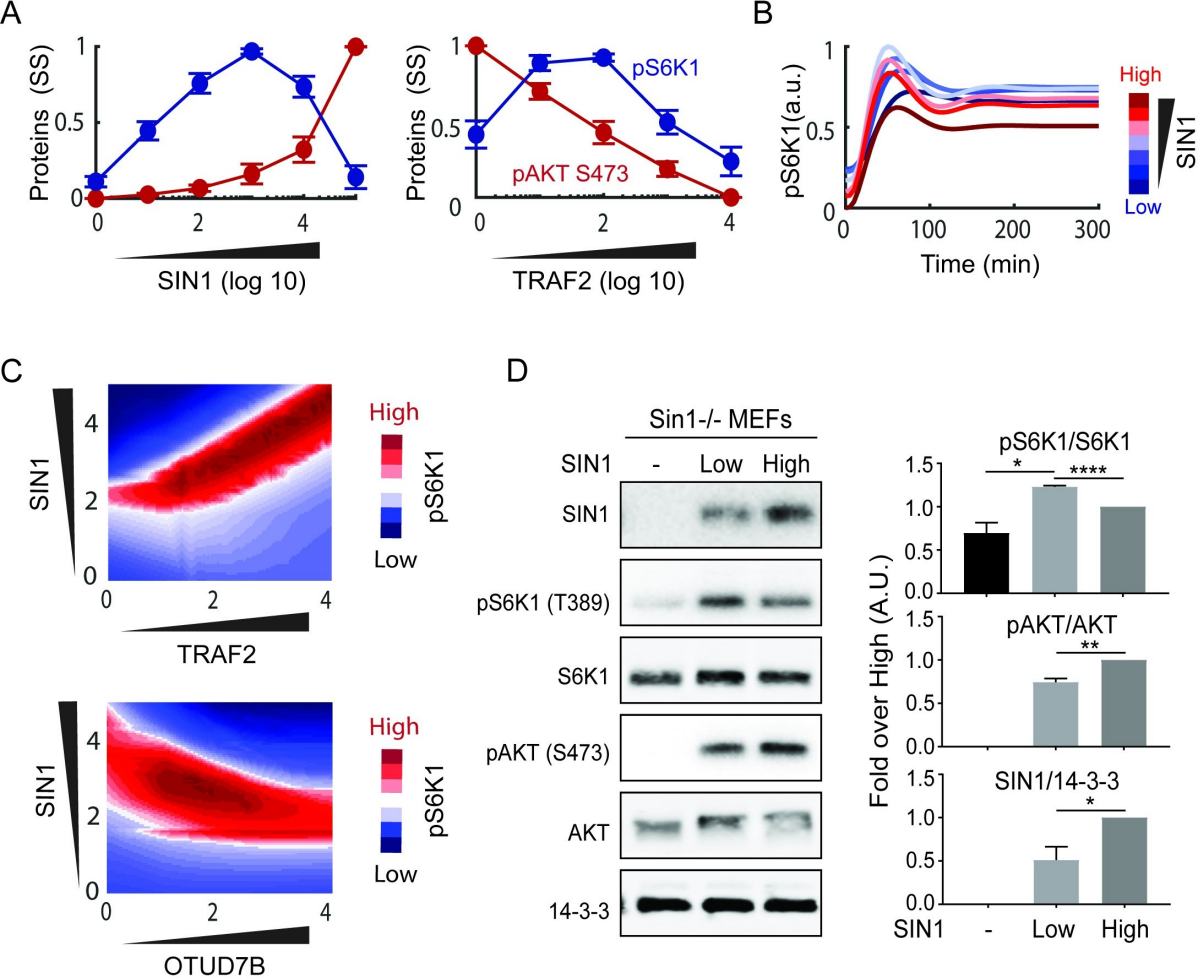
In silico analyses and experimental validation show biphasic dependency of mTORC1 activity on SIN1. (A) Dose-response simulations of the steady-state values of pS6K1 and pAKT S473 in response to increasing SIN1 or TRAF2, revealing a biphasic dependence of mTORC1 activity on SIN1. SIN1 or TRAF2 concentrations were varied within a wide range: 100 folds down and 1000 folds up of their nominal values. Each curve related to each parameter set was normalized by its maximal (peak) value and then average and S.E.M. were calculated at each concentration. (B) Temporal simulations of pS6K1 at different concentrations of SIN1. (C) Two-dimensional perturbation analysis showing the simulated steady-state response of pS6K1 to simultaneous change in pairs of proteins, which demonstrates the biphasic pattern is robust over wide ranges of TRAF2 and OTUD7B expression levels. (D) Experimental validation of the SIN1-mTORC1 biphasic dependency. Cells were deprived of serum for 2 h, followed by insulin (100 nM) stimulation for 10 min, and samples were analyzed by Western blot. Graphs show mean ± S.E.M. of quantitative analyses of Western blots (*P<0.05, **P<0.01, ****P<0.0001, two-tailed student’s t test, n = 3 biological replicates).

Interestingly, model simulations predict that increasing SIN1 induces a biphasic, dose-dependent response in pS6K1 (Fig 4A). Time-course simulations in Fig 4B confirm that an increase of SIN1 from a low level initially promotes pS6K1 (first phase), but further increase of SIN1 beyond a critical threshold suppresses pS6K1 instead (second phase). Biphasic patterns, albeit to a lesser extent, are also observed for TRAF2, raptor and OTUD7B graded perturbations (Figs 4A and S4). The underlying mechanism for SIN1-induced biphasic pattern can be explained based on the fact that MLST8 is competitively sequestered by raptor and SIN1 for assembly of mTORC1 and 2, respectively. According to Eq (1), the overall ability of mTORC1 to phosphorylate S6K1 is determined by two factors: (i) the abundance of mTORC1 as well as (ii) the kinase activity potential per mTORC1 molecule, the latter is proportionally dependent on the upstream kinases AKT and mTORC2. Thus, the level of pS6K1 is dictated by the balance between these factors. During the first phase, an increase in SIN1 would sequester more MLST8 and increase mTORC2 formation, leading to higher mTORC1 activity potential, but at the same time resulting in less mTORC1 formation. As the gain in mTORC1 activity outweighs the loss in abundance, the net effect is an overall enhancement of pS6K1. In contrast, the balance is tipped in the opposite direction in the second phase, as a further increase of SIN1 reduces mTORC1 abundance dramatically, which overrides the upregulation in mTORC1 activity, leading to an overall reduction in pS6K1.

To further investigate the robustness of this biphasic dependency with regard to changes in the expression of multiple proteins, we extended the sensitivity analysis to two dimensions by assessing the level of pS6K1in response to simultaneous changes in the abundance of pairs of state variables. Fig 4C shows that the SIN1-dependent pS6K1 biphasic response is present over a large range of TRAF2 expression levels. Similarly, the biphasic response persists over a wide range of OTUD7B expression levels. Together, these simulation results indicate that the biphasic mTORC1 activation induced by SIN1 is robust to expression variation in multiple network components.

Unlike pS6K1, model simulation predicts a monotonic increase of pAKT S473 in response to SIN1 overexpression (Fig 4A). Similarly, increasing OTUD7B and TRAF2 concentration monotonically promotes and diminishes pAKT S473, respectively; owing to their opposing effects on deubiquitinated MLST8 and mTORC2 formation (Figs 4A and S4). Interestingly, simulations show that overexpression of MLST8 enhances the level of both pAKT S473 and pS6K1 (S4 Fig), suggesting MLST8 promotes activation of both MTOR complexes. The model prediction is consistent with previous findings that MLST8 is upregulated in several cancer types [13]. Further, knockdown of MLST8 suppresses tumor growth by inhibiting MTOR complex formation and activity [13].

### Experimental validation of SIN1-induced biphasic mTORC1 dependency

In order to experimentally validate the predicted biphasic dependency of mTORC1 on SIN1, we generated MEF cells that express increasing levels of SIN1. To this end, we utilized MEFs where SIN1 has been knocked out and transfected these with the SIN1 construct containing EGFP as a sorting marker. The SIN1 low- and high-expression cells were sorted according to the expression level of EGFP. To measure the effect of graded SIN1 levels on phosphorylated S6K1, cells were stimulated with insulin after serum starvation and pS6K1 was measured using Western Blot (Fig 4D). In cells with no SIN1, there was a low level of pS6K1, which results primarily from pAKT T308 activity alone since mTORC2 is not functional in these cells, evident by the lack of any pAKT S473 signal (Fig 4D). In cells with low SIN1, there was a significant increase in the level of both pS6K1 and pAKT S473, the latter due to the formation of functional mTORC2. However, in cells having the highest levels of SIN1, while pAKT S473 was further increased, the level of pS6K1 was instead significantly reduced in comparison to SIN1-low cells (Fig 4D). These data confirm the model predictions and demonstrate a biphasic dependence of mTORC1 activity on SIN1 expression. Furthermore, in contrast to the results in previous studies indicating SIN1 knockout and mTORC2 activity have no effects on mTORC1 function [9,10], our model simulations verified by experimental data here indicate that mTORC2 regulates both the activity of AKT and mTORC1 in MEF cells.

### Interrogating the SIN1-mTORC1 biphasic dependency in diverse cancer cell lines

By integrating model-based simulation and biological validation, we have identified a previously unknown biphasic dependency between SIN1 and mTORC1 activity in MEF cells. To investigate this biphasic connection under various cellular contexts and how it may be impacted by cell-to-cell variability in protein expression, we adjusted our model by incorporating cell-type specific protein expression data from diverse cell types and performed simulations under these varying conditions.

**Fig 5:**
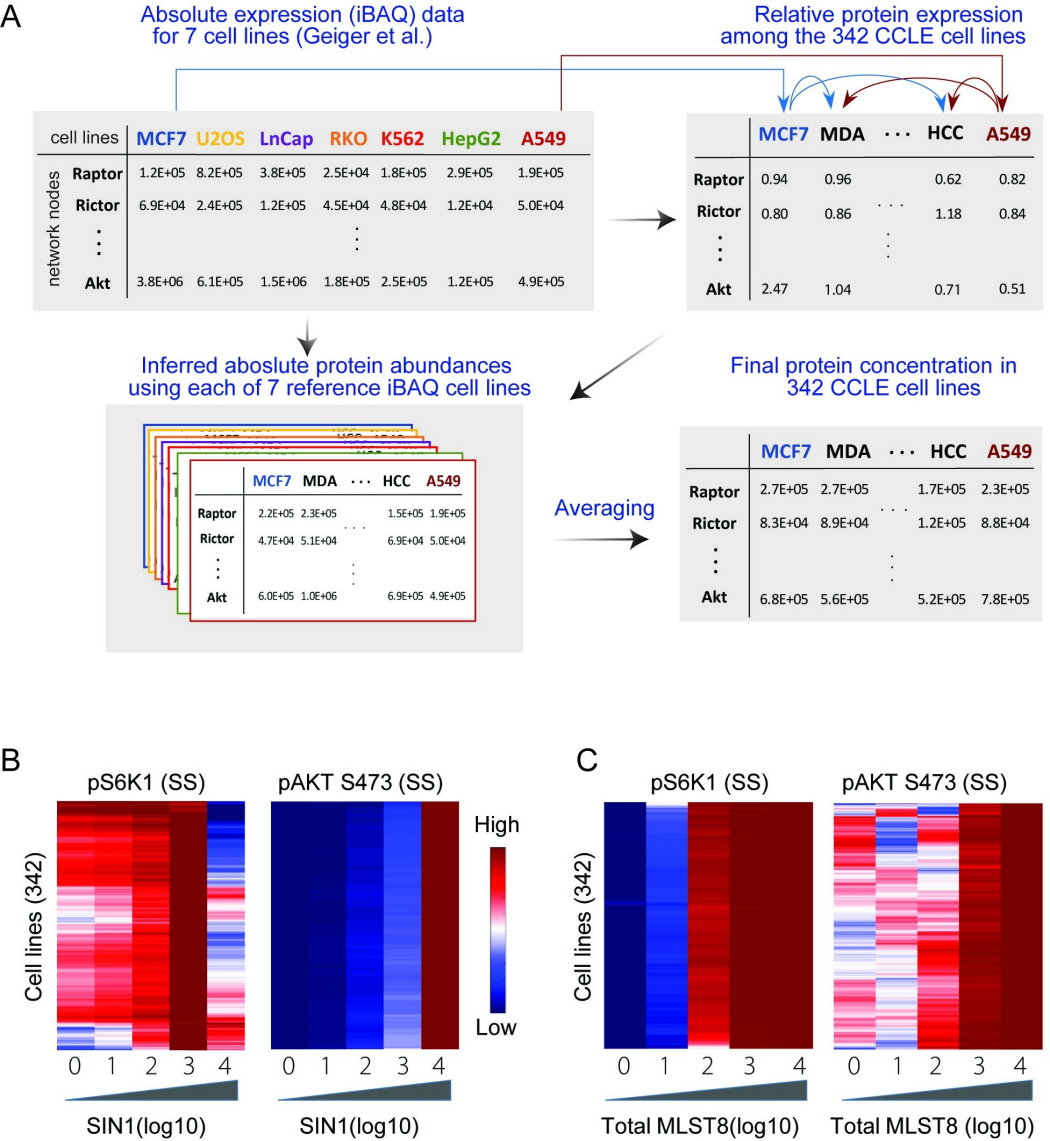
The mTORC1-SIN1 biphasic dependency is robust under diverse cellular contexts. (A) Pipeline for inference of absolute protein levels in 342 CCLE cell lines by combining CCLE relative expression and Geiger iBAQ-based absolute expression data. (B) Heatmap representing dose-response simulation of pS6K1 in response to increasing SIN1 expression, showing the SIN1-mTORC1 dependency is a robust biphasic pattern across multiple cell lines. For each line, SIN1 concentration is perturbed from 0.01 to 1000 fold of its initial value and pS6K1 is measured at the steady state (SS). (C) Heatmaps representing dose-response simulation of pS6K1 (left) and pAKT S473 (right) in response to increasing MLST8 expression in 342 cancer cell lines.

To this end, we first obtained *relative* protein expression recently reported by the Cancer Cell Line Encyclopedia (CCLE) for 375 human cancer cell lines [33], which allowed us to compare protein levels *across* the various cell lines. Of these, 33 cell lines have missing data, i.e. no detectable expression of one of the model proteins, leaving 342 for further analysis. However, tailoring our model to a new cell type ideally requires *absolute* protein levels, i.e. abundance of proteins *within* a proteome. To address this, we utilized a second dataset containing absolute protein abundances obtained by Geiger et al. [34] for 11 common cell lines, using mass spectrometry based label-free proteomics and intensity-based absolute quantification (iBAQ) algorithm [34]. Since 7 cell lines were consistent between these two datasets, we could use the iBAQ data of these 7 cell lines and the relative protein information in 342 cell lines to infer the absolute protein levels for the CCLE cohort (see Material and Methods for detailed description). A schematic of this inference pipeline is illustrated in Fig 5A. Using MCF7 as an example, we combined absolute protein abundances (i.e. iBAQ data) in MCF7 and the relative expression data (i.e. CCLE data) between MCF7 and 341 remaining cell lines to compute the absolute protein abundances for these cells. We repeated this process for the 6 remaining cell lines with iBAQ data (Fig 5A). As a result, for each of the 342 CCLE cell lines we have 7 *sets* of protein abundances that were inferred using each of the 7 cell lines from [33]. The final abundance of the model protein components in each cell line were then derived by taking average of the 7 corresponding values, which were subsequently used to modify the initial conditions in our model in order to tailor it for each of the 342 cancer cell lines (see Material and Methods for detailed description).

Having customized our model for different cell lines, we asked if the SIN1-mTORC1 biphasic dependency still exists under these varying cellular contexts. Fig 5B displays the model simulation results for pS6K1 in response to increasing SIN1 expression, showing the biphasic dependency is still present in almost all of the cell lines. This suggests the biphasic dependency is robust to intercellular variation, albeit the precise shape of this biphasic curve differs between different cell types, where it peaks at a lower level of SIN1 in some compared to others (Fig 5B). On the other hand, increasing SIN1 consistently promotes phosphorylated AKT levels in all the cell types (Fig 5B). In addition, we analyzed the effect of MLST8 perturbation in various network conditions (Fig 5C). In contrast to SIN1, increasing MLST8 consistently enhanced the levels of phosphorylated S6K1 and AKT in the majority of the tested cell types. These results highlight the role of MLST8 overexpression in tumor progression in part through promoting mTORC1 and 2 activities, which was observed in colon carcinoma and prostate cancer [13].

### Molecular factors governing the SIN1-mTORC1 biphasic dependency

**Fig 6:**
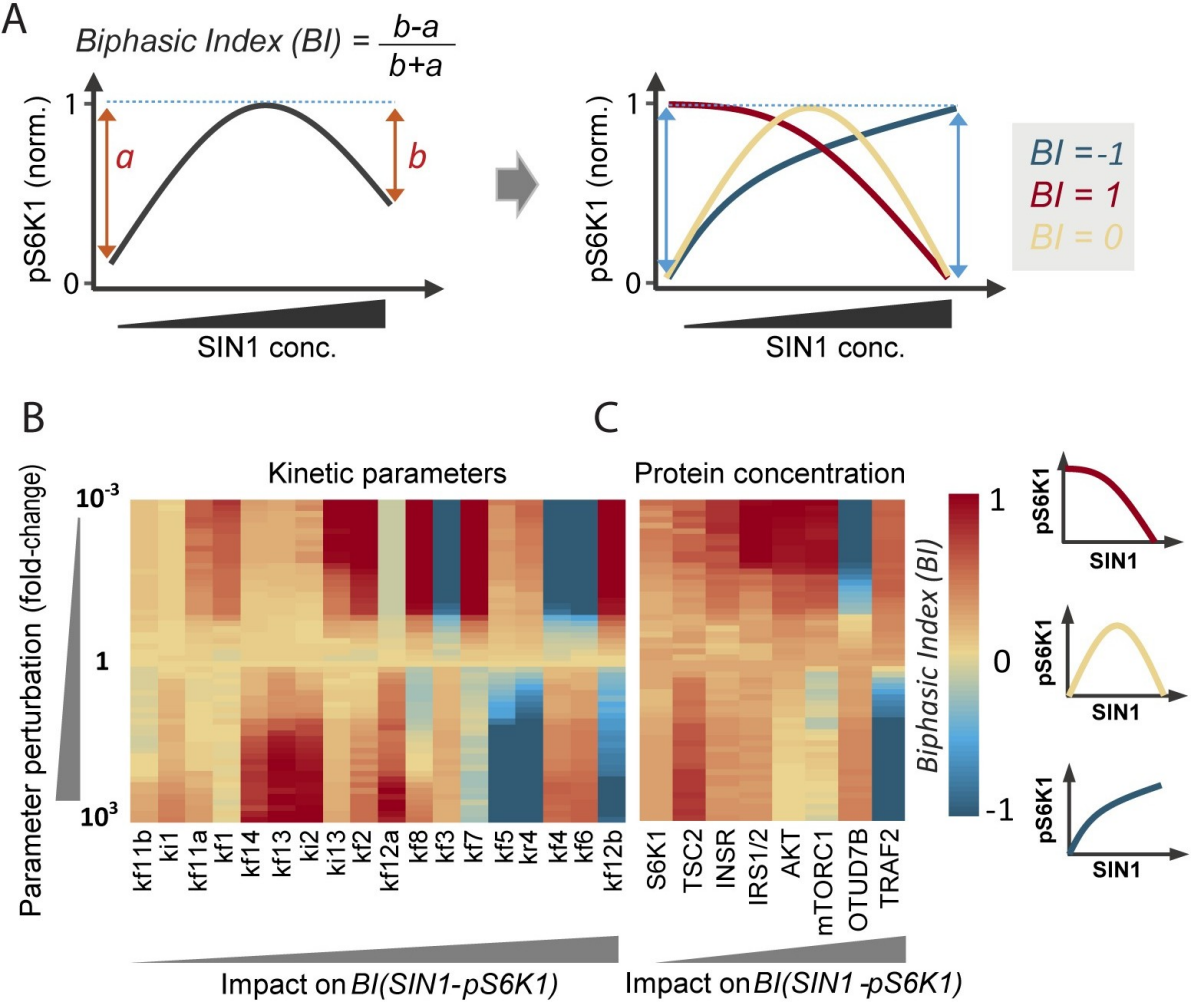
Deciphering the molecular players that govern the SIN1-mTORC1 biphasic dependency. (A) Derivation of a quantitative Biphasic Index (BI) measuring the *biphasicness* of pS6K1 response curve to SIN1 perturbation. To generate the SIN1-pS6K1response curve for a specified parameter set, we gradually increase SIN1 expression within the range = [0.01, 1000] folds of the nominal value and we simulated the corresponding pS6K1 level and obtained its steady state value. The response curve is then normalized to its peak (max) value. BI ranges between -1 and 1: BI = -1 or 1 indicate a strictly monotonic increasing (blue) and decreasing response curve (red), respectively; while -1< BI <1 indicates a biphasic pattern that is more pronounced as BI is closer to 0 (yellow). (B-C) Impact of perturbation of model kinetic parameters (B) or protein concentrations (C) on the BI of the SIN1-mTORC1 response curve. The kinetic parameters were perturbed one by one within wide ranges (1000 folds up/down of nominal values), and the impact on the BI were computed. For the analysis all the best-fitted parameter sets were used for the parameter perturbation analysis and the average results across these multiple parameter sets were calculated. The parameters/proteins are ranked as having minimum (left) to maximum (right) effect on variation of BI (SIN1-mTORC1).

A strength of a modelling approach is that potential factors controlling the emergent complex network behaviors can be systematically tested through *in silico* perturbation/sensitivity analysis, which would otherwise be challenging experimentally. Here, we seek to decipher the molecular players that govern the observed SIN1-mTORC1 biphasic dependency. To evaluate a biphasic response quantitatively, we introduced a general ‘biphasic index’ (BI) that measures the *biphasicness* of a response curve, as defined in Fig 6A. Since the response curve is normalized to its peak (maximal) value, BI ranges between -1 and 1. BI = -1 or 1 indicate a strictly monotonic increasing or decreasing response curve, respectively; while -1< BI <1 indicates a biphasic pattern that becomes more pronounced as BI is closer to 0 (Fig 6A). Next, we systematically perturbed the model kinetic parameters representing the strength of network interactions one by one within wide ranges (1000 folds up/down of nominal values) and assessed the impact of these perturbations on the BI of the SIN1-mTORC1 response curve.

The results, displayed in Fig 6B, indicate that the parameters related to the MLST8 switch: (de)ubiquitination of MLST8 (*k*_*f4*_ and *k*_*r4*_), MLST8-SIN1/raptor binding (*k*_*f6*_/*k*_*f5*_), and OTUD7B activation (*k*_*f3*_) have the strongest impact on the shape of the SIN1-pS6K1response curve (S5 Fig). Indeed, lowering *k*_*f3*,*4*,*6*_ converts the SIN1-pS6K1curve from biphasic to monotonic increasing, while raising them shifts the curve to a monotonic decreasing pattern instead (the opposite is true for *k*_*f5*_ and *k*_*r4*_). Consequently, the biphasic response exists over relatively restricted ranges of these parameters (yellow regions, Fig 6B). In contrast, the biphasic response persists over much wider ranges of the lower-ranked parameters, and their perturbations largely shifts the curve to an increasing pattern only (yellow to red, Fig 6B), suggesting the biphasic response is less sensitive to changes in the switch non-related parameters. Of note, the lowest-ranked parameters (*k*_*f11b*_, *k*_*i1*_) do not significantly affect the biphasic pattern. Interestingly, the sensitivity analysis further reveals that *k*_*f12a*_ and *k*_*f12b*_ impact the BI in opposite ways, indicating the rate of TSC2 phosphorylation by pAKT T308 (*k*_*f12a*_) or the fully activated ppAKT (*k*_*f12b*_) have divergent influence on the SIN1-pS6K1response. The reason for this is that ppAKT is regulated by mTORC2 activity and therefore SIN1 concentration, whereas pAKT T308 is independent from SIN1.

In addition, we performed similar sensitivity analysis for the model state variables (i.e. proteins’ concentration). Fig 6C shows that in accordance with the results above, perturbing OTUD7B and TRAF2, the primary regulators of the MLST8 (de)ubiquitination switch, most strongly affect the BI of the SIN1-pS6K1 response curve. Collectively, these results indicate that the MLST8 ubiquitination switch and its constituents critically govern the biphasic relationship between mTORC1 activity and SIN1 concentration.

### MLST8 represents a viable therapeutic target in breast cancer

**Fig 7:**
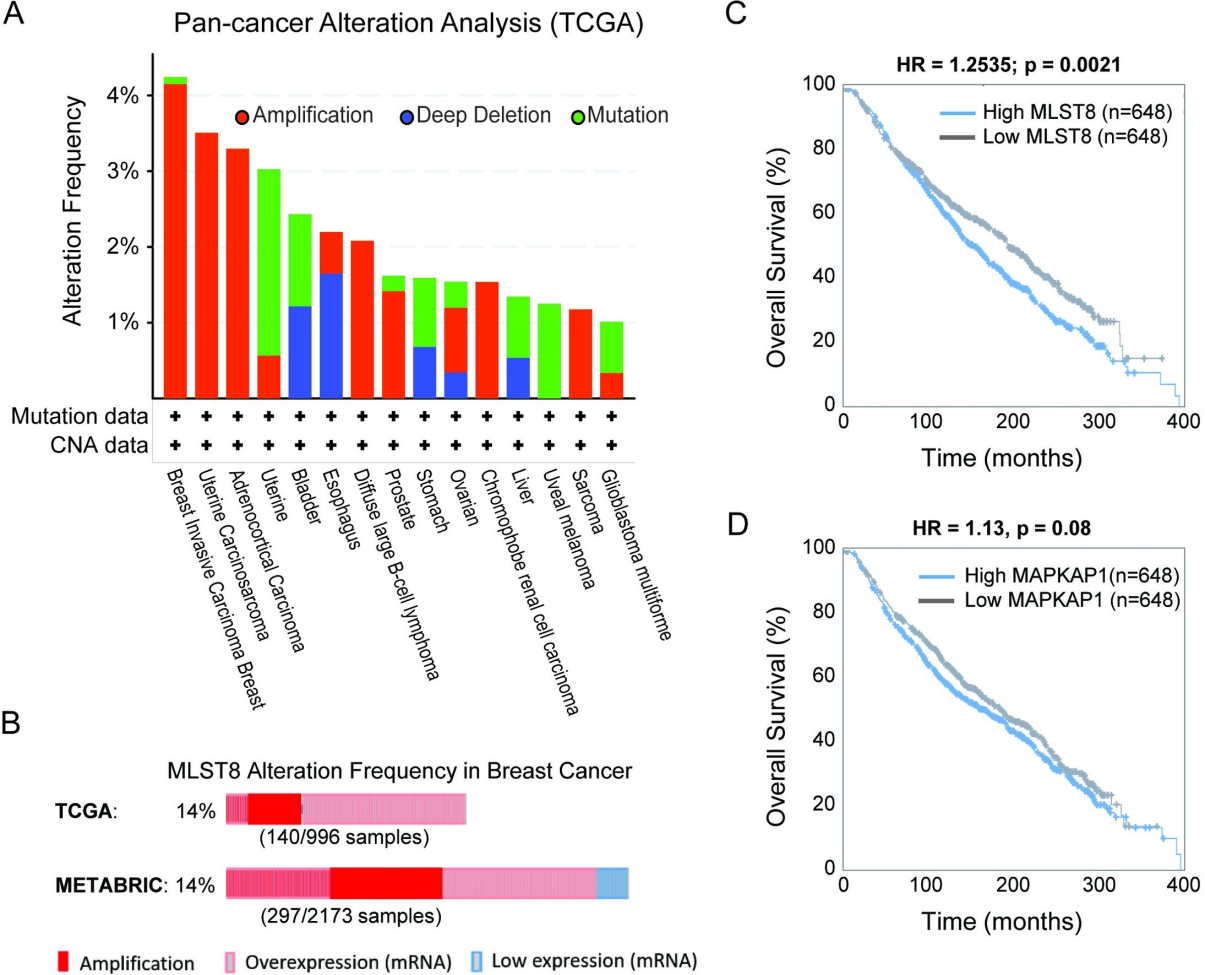
Interrogation of MLST8 alteration and prognostic value in cancer patients. (A) Alteration frequency of the MLST8 gene in cancer patients analyzed from TCGA database using cBioPortal (www.cbioportal.org), shown for the most frequently altered tumour types (cut-off > 1%). (B) Frequency of MLST8 alteration in breast cancer patients analyzed using two breast cancer cohorts TCGA and METABRIC. Only patients having MLST8 alterations are displayed for brevity. (C) Breast cancer patients with high MLST8 expression are significantly associated with poorer overall survival outcome compared to those with low MLST8 expression. (D) No significant differences in overall survival between patients having ‘low’ and ‘high’ expression of MAPKAP1, the gene encoding SIN1.

Our model simulation showed that MLST8 enhances the activity of both mTORC1 and 2 (Figs 5C and S4), suggesting it plays a tumour-promoting role. To examine this further, we interrogated the alteration profiles of MLST8 in cancer patients from The Cancer Genome Atlas (TCGA). In line with our prediction, MLST8 is primarily amplified in the top frequently-altered tumour types, including breast cancer, uterine carcinosarcoma and adrenocortical carcinoma (Fig 7A). Further analysis of breast cancer, where MLST8 is most commonly amplified, shows that it is frequently overexpressed (~14%) in patients from both the TCGA and METABRIC cohorts (Fig 7B), two of the largest publicly-available breast cancer cohorts. Importantly, survival analysis demonstrates that high MLST8 expression is significantly associated with poorer overall survival in breast cancer patients (Fig 7C). Unlike MLST8, there are no differences in the overall survival of patients with low and high expression of MAPKAP1, the gene encoding SIN1 (Fig 7D). This is in line with the biphasic feature of the SIN1-pS6K1activity response, where low or high SIN1 lead to comparable levels of phosphorylated S6K, a marker for tumour cell survival. Together, these results indicate MLST8 plays a tumour-enhancing role in breast cancer and its expression may serve as a prognosis indicator, in line with its reported role in other tumour types (12). These findings also point to the exciting possibility of targeting MLST8 as a future therapeutic intervention strategy in breast cancer.

## Discussion

Signalling networks are the key information-processing machineries that underpin the ability of living cells to respond proportionately to extra- (and intra-) cellular cues. Studies of these networks reveals they are complex interaction systems, often consisting of multiple feedback mechanisms, post-translational modifications and intricate pathway crosstalk. In-depth understanding of these networks thus requires quantitative approaches that complement biological experimentation. In this study, we integrated predictive modelling and experimental studies to characterize the emergent dynamic properties of the PI3K/MTOR signalling network, a central regulator of cellular growth, aging and metabolism.

Dynamic modelling has been extensively employed in the study of cell signalling [35]. Here, we present new dynamic models of the PI3K/MTOR network that explicitly accounts for the critical coordination of mTORC1 and 2 formation and function by (de)ubiquitination of the shared subunit MLST8 [7]. To our knowledge, these are the first models to do so. We initially postulated four different network structures and corresponding model variants (models 1–4) that reflect competing hypotheses on OTUD7B regulation and whether MLST8 is essential for mTORC1 functional activity. The latter remains a contentious point among several published studies [9–13]. By validating model simulations against multiple experimental datasets, we were able to discriminate among the alternative network structures and arrived at model 4 as the most likely model as it provides the best fit to the data. Importantly, this model was subsequently validated by new biological experiments. Collectively, these integrative analyses demonstrate that MLST8 is required for the assembly and activity of mTORC1, and the deubiquitinase OTUD7B is stimulated through IRS1/2 rather than INSR directly (Fig 3).

Although model 4 best fitted the data among the considered models variants, it did not perfectly recapitulate the quantitative dynamic features of certain network nodes, e.g. mTORC2 and pTSC2 under TRAF2-deleted condition (Fig 2D). There are several possible reasons for such discrepancy. First, while we aimed to include the salient regulatory mechanisms within the insulin/PI3K/MTOR network, model 4 remains an abstraction of reality and a simplified version of the real network. Second, as the involvement of TRAF2 in PI3K/MTOR signalling has only been recently reported [7], we still lack a full mechanistic understanding of its role in regulating the network behaviour. Since TRAF2 has diverse functions [36], it may have unknown regulation towards pTSC2 that is independent of MLST8. Future incorporation of these potential mechanisms will likely further improve the quality of model 4.

Using model 4 for subsequent analysis, model simulations predicted a hitherto unknown biphasic dependency between mTORC1 activity and SIN1, a central subunit of mTORC2. Importantly, we confirmed this prediction experimentally by gradually overexpressing SIN1 in SIN1-/- MEF cells and measured phosphorylated S6K1 and AKT (pS473) as indicators of mTORC1 and 2 activity, respectively. Indeed, increasing SIN1 concentration from a low level significantly enhanced mTORC1 activity. However, beyond a critical threshold, further overexpression of SIN1 inhibited mTORC1 activity (Fig 4A and 4D). This is in contrast to the monotonic increase in mTORC2 activation in response to SIN1 overexpression observed in both model simulations and experimental data. To identify the molecular factors that control the SIN1-mTORC1 biphasic response, we performed model-based sensitivity analyses assessing possible effects of changes in model kinetic parameters and state variables on the *biphasicness* of the response curve, quantified by a newly derived metric. Interestingly, we found that the top-ranked, i.e. most dominant, parameters and state variables are primarily involved in the regulation of the MLST8 (de)ubiquitination switch, suggesting this molecular mechanism plays an important role in modulating the biphasic level of the SIN1-mTORC1 curve. In line with this finding, the mechanistic explanation underlying the biphasic response stems from the switch-mediated protein competitions and the balance of such competitions that play out within the network. Increasing SIN1 sequesters MLST8 from raptor to form more mTORC2 and less mTORC1 at the same time; however higher levels of mTORC2 promotes stronger AKT activation, which converts more mTORC1 molecules from inactive to active state. In the first phase, the gain in mTORC1 activity dominates the loss in abundance, and the net effect is an enhancement of pS6K1, while the balance is flipped in the second phase, leading to an overall reduction in pS6K1.

While our integrative analyses demonstrated the biphasic SIN1-pS6K1dependency using MEF cells as the main experimental model, we wondered whether such response is robust to cell-to-cell variability and if it is conserved under different cellular contexts. Given the relevance of the PI3K/MTOR signalling network in cancer and availability of large-scale protein expression data in cancer cell lines, we integrated relative and absolute protein abundances from complementary proteomics studies and developed a pipeline to infer absolute protein abundances for 342 cancer cell lines within the CCLE consortium. Using these data, we adjusted our model for each individual cell type by changing the total expression of model proteins (i.e. initial conditions) accordingly, and simulated the SIN1-pS6K1response curve for each cell type. Simulation results predict that while the precise shape of the response curve vary between the cell types, the biphasic pattern is still present in most of them, suggesting the biphasic dependency is robust to the cell-to-cell heterogeneity in protein expression.

This biphasic (rather than monotonic) pattern may underlie the diverse and inconsistent response of phosphorylated S6K1 to mTORC2 inhibition reported by different experimental studies. For example, blocking mTORC2 reduced pS6K1 in breast cancer MCF7 and ZR-75-1 cells [37] as well as in non-transformed cells, including 3T3-L1 [26,38] and HEK-293 cells (3); whereas it enhanced pS6K1 in lung cancer A549 and prostate cancer PC3 cells (35); and induced no changes in pS6K1 in other tumour cells, including HT29 [39] and U251 [40]. Moreover, inconsistent observations were recorded even in the same cell line by different studies, e.g. as in the case of Hela [3,16] and MEF cells [9,10]. One reason for these discrepancies may be due to the non-linear feature of biphasic input-output curves, where depending on the starting value of the input (dose) and efficiency of knockdown/inhibition, this could either inhibit, promote or not affect the output (response) (see S6 Fig).

Another consequence of the SIN1-pS6K1 biphasic response pattern is that the level of pS6K1, which is indicative of mTORC1 activity, is maximized over an intermediate range of SIN1 expression. Since the biphasic pattern was found to be present across many different cancer cell types, this may provide a conserved mechanism for tumour cells to sustain proliferative and survival signalling elicited by mTORC1 against possible variation in SIN1 expression, which can arise from the molecular heterogeneity within a cell population [41]. This property is analogous to the biphasic response conferred by scaffold proteins to signalling cascades, where signalling outputs can be fine-tuned by the expression level of the scaffolds [42,43]. The existence of a biphasic relationship among network components further highlights the nonlinear dependency of signalling outputs on the network conditions, emphasizing the need to embrace biological contexts while studying cell signalling. Indeed, depending on the specific expression of SIN1, this could lead to entirely different activation levels of mTORC1. This notion is in contrary to the linear viewpoint that is still prevalent in cell signalling cascades, where an ‘upstream’ node is often assumed to have a linear (either positive or negative) effect on a downstream node regardless of the specific network context. In addition, as SIN1 represents a potential therapeutic target for cancer therapy (3), the biphasic response of pS6K1 on SIN1 may have implication in the development of anti-SIN1 therapy. This is because therapeutic inhibition of SIN1 in SIN1-high tumour cells may inadvertently upregulate pS6K1 and promote tumour cell growth.

In addition to revealing a novel functional connection between mTORC2 and mTORC1, our model simulations further showed that MLST8 promotes the activity of both complexes (Figs 5C and S4). This result is consistent with the alteration profiles found in cancer patients analysed from TCGA, which show MLST8 is primarily amplified and overexpressed in the most frequently altered tumour types, notably breast cancer (Fig 7A and 7B). Model simulations further suggest that MLST8 may stimulate tumour development and/or progression through promoted activation of both MTOR complexes, a notion in line with published data in colon and prostate cancer [13] and our result that high MLST8 is associated with poorer overall survival in breast cancer patients (Fig 7C). Together, these findings highlight the potential of targeting MLST8 as an anti-cancer therapeutic strategy, which is currently under investigation [44].

In this study we considered SIN1 phosphorylation by AKT as the main mechanism for mTORC2 activation [5]. However, there is another potential activation mechanism for mTORC2 that is mediated by PIP3 [45]. To investigate whether the AKT-mTORC2 positive feedback loop is required to reflect the experimental observations, we modified model 4 by removing the link from AKT to mTORC2 and instead assumed that mTORC2 can be activated by PIP3. As PIP3 was not explicitly included in our model for the sake of simplicity, we assumed mTORC2 is activated by the upstream component pIRS1/2, and re-calibrated the modified model against the same data. Simulation results of the modified model compared to the experimental data shows there is no significant difference in fitting quality between the modified and the original model 4 (see S7 Fig). Together, this suggests that the AKT-mTORC2 positive feedback loop is not required to reflect the experimental data, but also does not harm the simulation results.

In conclusion, we have integrated mechanistic modelling and experimental analysis to elucidate novel emergent behavior of the PI3K/MTOR signalling network. In contrast to the commonly-held view that mTORC2 lies upstream and is a positive regulator of mTORC1, we found that their relationship is highly non-linear. Our results highlight the need to embrace network-level view and integrative approaches for the study of complex signalling systems. The new model developed here is the first that incorporates the MLST8 ubiquitination switch, and provides a useful quantitative framework for future studies of PI3K/MTOR signalling.

## Material and methods

### Cell culture and viral transduction

PlatE cells were cultured in DMEM with 10% FBS, 2 mM L-GlutaMAX, 1 μg/ml puromycin and 10 μg/ml blastcidine. SIN1-/- MEFs and C2C12 myoblast cells were cultured in DMEM with 10% FBS, 2 mM L-GlutaMAX, non-essential amino acids, and 1 mM sodium pyrophosphate.

For SIN1 retroviral production, PlatE cells [34] grown in 10 cm dish were transiently transfected using lipofectamine 2000 (Life Technologies) according to manufacturer’s instructions with retroviral vectors pMIG or pMIG-SIN1. Medium was replaced the next day with 6 ml medium per dish. Virus-containing medium was collected after 2 days, followed by filtration using a 0.45 micron filter and used immediately for infection or stored at -80°C.

To generate SIN1 re-expression MEFs, SIN1-/- MEFs were seeded into retroviral-containing media and transduced overnight with 4 μg/ml polybrene, followed by fresh media change the next morning.

The SIN1 low- and high-expression cells were sorted by FACS (FACSAria II) according to the expression level of EGFP.

### Plasmids, siRNA and transfection

pLentiCMV-Blast MLST8-Flag was a gift from Jin Chen (Addgene plasmid # 124915; http://n2t.net/addgene:124915; RRID:Addgene_124915). MISSION esiRNA targeting Renilla Luciferase (EHURLUC), mouse IRS1 (EMU061331) and IRS2 (EMU187631) were purchased from Sigma.

C2C12 myoblast cells were transfected with siRNA at approximately 70–75% confluency using *Trans*IT-X2 (Mirus Bio) according to manufacturer’s instructions. Twenty-four hours later, cells were transfected with HA-ubiquitin and MLST8-Flag using Lipofectamine 3000 (Life Technologies) according to manufacturer’s instructions. Cells were serum starved for 16 hours followed by insulin stimulation (100 nM, 15 min), and harvested for immunoprecipitation 48 h post-transfection.

### Immunoprecipitation

Whole-cell lysates were prepared in Flag IP buffer (1% NP-40, 10% glycerol, 150 mM NaCl, 50 mM Tris-Cl pH 7.4) supplemented with EDTA-free protease inhibitors and phosphatase inhibitors. The total protein concentrations of whole-cell lysates were measured by the FLUOstar Omega microplate reader (BMG LABTECH) using BCA assay reagent. Lysates were incubated with Flag antibody overnight at 4°C followed by 1hour incubation with protein G beads (GE Healthcare). Immunoprecipitates were washed three times with IP buffer followed twice washing with PBS buffer before being resolved by SDS–PAGE and immunoblotted with indicated antibodies.

### Western blotting

Samples were separated by SDS–PAGE and transferred to PVDF membranes. The membranes were incubated in a blocking buffer containing 5% skim milk in Tris-buffered saline (TBS) and immunoblotted with the relevant antibodies overnight at 4°C in the blocking buffer containing 5% BSA–0.1% Tween in TBS buffer. After incubation, the membranes were washed and incubated with HRP-labeled secondary antibodies for 1 h and then detected by SuperSignal West Pico Chemiluminescent Substrate. In some cases, IR dye 800-conjugated secondary antibodies were used and then scanned at the 800-nm channels using an Odyssey IR imager. Immunoblots were quantified by Image studio software and statistical significance was assessed using Student’s t test.

The following antibodies were used: SIN1 (Millipore, 07–2276), S6K1(CST, 2708), S6K-T389 (CST, 9234), AKT (CST, 4051), AKT-S473 (CST, 4058), AKT-T308 (CST, 9275), PRAS40 (CST, 2691), PRAS40-T246 (CST, 2997), IRS1 (CST, 3407), IRS2 (CST, 3089), MLST8 (CST, 3274), HA (CST, 3724)) and 14-3-3 (Santa Cruz, sc-629).

### Mathematical modelling and parameter estimation

We constructed four mechanistic models to interrogate different possible network structures of the PI3K/MTOR signalling pathway. With these models we examined whether: (*i*) The OTUD7B activity is regulated by the S6K1 negative feedback in the pathway, and (*ii*) MLST8 is required for mTORC1 kinase activity. With these four models we assessed the 4 different possible combinations. The models are constructed using ordinary differential equations (ODEs). The ODEs and the best-fitted parameter sets for each model are given in S1 and S2 Tables. The model formulation and calibration processes were implemented in MATLAB (The MathWorks. Inc. 2019a).The IQM toolbox (http://www.intiquan.com/intiquan-tools/) was used to compile the IQM file for a MEX file which makes the simulation much faster.

Model training is the process of estimation of the model’s parameters. As a result of model calibration a ‘best-fitted’ model will be produced that best recapitulates biological data used for model training. To calibrate the model parameters objective function *J* was used that quantifies the difference between the model simulation results and corresponding experimental measurements:

$$J(p)=\sum_{j=1}^{M}\sum_{i=1}^{N}({y_{j,i}^{D}-y_{j}(t_{i},p))}^{2}$$

The model parameters value were estimated to minimize the objective function value. Here, *M* is the number of experimental data sets for fitting and *N* denotes the number of time points in a experimental data set. *y_j_*(*t_i_, p*) indicates the model simulations of the component *j* at the time point *t_i_* while parameter set *p* is used for the simulation. Finally, $y_{j,i}^{D}$ is the experimental data of component *j* at time point *t_i_* and *w_j_* is the weight of the component *j*.

For each model, parameter estimation was undertaken using a genetic algorithm (GA)-based optimization procedure (Man et al., 1996; Reali et al., 2017; Shin et al., 2014). For this, the Global Optimization Toolbox and the *ga* function in MATLAB were used. The GA runs were implemented with population size set to 200 and the generation number set to 800. Because there likely exist multiple optimal parameter sets that fit the experimental data more or less equally well, we repeated the GA procedure 500 independent times for each model in order to identify as many best-fitted parameter sets as possible for subsequent ensemble simulations. Selection of these best-fitted sets for further analysis was based on two key criteria: (i) first, the objective function values (i.e. the numeric measures of the aggregated discrepancy between the simulated and experimental values) for each of these sets must be under a cut-off threshold, as shown in S8 Fig; and (ii) second, they should also pass a qualitative assessment based on visual inspection of how well the simulated curves match the experimental data curves. The reason for including the latter is because sometimes a fitted parameter set can yield a low objective function value but does not display good qualitative fitting. Combination of both quantitative and qualitative assessments allowed us to arrive at high-confident best-fitted parameter sets for each model. As a result, we selected 189, 198, 12, 13 best-fitted parameter sets for Model 1, 2, 3 and 4 for further simulation and analysis, respectively. The same cut-off threshold for the objective function value was applied across the models and determined based on visual assessment of the fitting quality.

To examine the similarity between the chosen best-fitted parameter sets for model 4, we performed hierarchical clustering analysis of these sets. S9 Fig shows that the parameter sets were quite heterogeneous and poorly clustered, suggesting the model is non-identifiable as expected and our genetic algorithm has managed to unbiasedly explore a diverse landscape of local minima in the high-dimensional parameter space, again highlighting the need to consider multiple best-fitted sets.

### Inference of protein concentrations for model customization

We inferred the absolute protein abundance of the proteins of interest (in this case the network nodes) for each of the 342 cancer cell lines in the CCLE database according to the following formula:

$$Protein\;Concentration\;(i,x)=\frac{1}{7}\sum_{j=1}^{7}IBAQ(i,j)\times \frac{CCLE(i,x)}{CCLE(i,j)}=\frac{\frac{IBAQ(i,MCF7)}{CCLE(i,MCF7)}+\frac{IBAQ(i,U2OS)}{CCLE(i,U2OS)}+\cdots +\frac{IBAQ(i,A549)}{CCLE(i,A549)}}{7}\times CCLE(i,x)$$

Here *i* and *x* denote protein *i* in cell line *x*, which is one of the 342 cell lines obtained from the CCLE database. *IBAQ(i*, *j)* denotes the absolute abundance (copies number) of protein *i* in cell line *j*, where *j* represents one of the seven cell lines obtained from the iBAQ dataset: *j* = (*MCF7, U2OS, LnCap, RKO, K*562, *HepG2* or *A*549); and *CCLE(i*, *j)* is the relative expression of protein *i* in cell line *j* obtained from the CCLE database.

S10 Fig displays the inferred concentration for the relevant proteins for four examples cell lines, which have been averaged across 7 values obtained by using each of the 7 reference cell lines. Here, error bars represent the standard errors across the 7 replicates. As we can see consistently between the proteins, the standard errors are relatively small indicating that the individual inferred values are similar along the 7 replicates.

Next, for each cell line we converted the protein abundance numbers into protein concentrations in nanomole (nM), which is the unit used in our models. For this, we utilised the following formula, which keeps the total protein concentration in each cell line to be the same as that in the MEF cell:

$$Protein\;Concentration\;(i,x)=\frac{Absolute\;abundance\;of\;protein\;(i)\;in\;cell\;line\;(x)\times 100\;(nM)}{Average\;of\;the\;absolute\;proteins\;concentration\;in\;cell\;line\;(x)}$$

### Sensitivity analysis

To investigate the molecular factors that govern the biphasic pattern between SIN1 concentration and mTORC1 activity we used sensitivity analysis. First, we defined a biphasic index (BI) that calculates the *biphasicness* of the pS6K1-SIN1 concentration response curve. For this, SIN1 concentration is perturbed from 0.01 to 1000 of its initial value and pS6K1 is measured to obtain the pS6K1-SIN1 concentration curve (Fig 6A). In the next step, this curve is normalized to 1 and BI index is calculated as defined in Fig 6A.

Next, value of each model kinetic parameter is perturbed from 0.001 to 1000 of its nominal value and the BI is calculated for each parameter value. Finally, the parameters are ranked based on their impact on variation of BI index after perturbing their value.

### mTORC1 total kinase activity

The kinase activity of the mTORC1 is defined as follows:

TotalmTORC1kinaseactivitypotential=(mTORC1abundances)×(activitypotentialperonemTORC1molecule) (1)

which indicates the total activity of mTORC1 is the product of the activity of each mTORC1 molecule and the abundance of mTORC1 in the cell.

### Patient data analysis

For survival analysis, mRNA expression and associated overall survival data for breast cancer patients from the METABRIC trial [46] were downloaded from the cBioPortal for Cancer Genomics portal (www.cbioportal.org). The patients were divided into 2 groups with ‘low’ (below the 34th percentile) and ‘high’ (above the 66th percentile) expression of the MLST8 (or MAPKAP1) gene. Accordingly, out of a total of 1904 cases available for analysis, 648 met the high-group criteria and 648 met the low-group criteria for inclusion into the analysis. Analysis comparing overall survival between these groups was implemented using a Log-rank test (with p< 0.05 considered significant). The Log-rank test statistics and survival curves were generated using Kaplan-Meier estimate and implemented using the Log rank package (https://www.github.com/dnafinder/logrank) in MATLAB 2019b.

## Supporting information

S1 Fig Quantified time -course data of various network components following insulin stimulation (100nM) in WT MEFs, reproduced from Western blot data representative of at least three biological replicates obtained from [7]. Phosphorylated (p) and ubiquitinated (Ubi) levels of each protein were normalized to the corresponding total protein levels. Each curve was normalized by their maximal (peak) value.
(TIF)

S2 Fig Quantified time-course data of the indicated proteins following EGF stimulation in WT and TRAF2 knocked-out MEF cells, reproduced from representative experimental data obtained from [7]. Phosphorylated (p) levels of each protein were normalized to the corresponding total protein levels. The curves under TRAF2 knockout condition were normalized by their corresponding maximal (peak) values, and the WT curves were scaled accordingly.
(TIF)

S3 Fig Visual depiction of signal flow within the PI3K/MTOR network. (A) In response to insulin stimulation, phosphorylated AKT increases. This leads to enhanced TSC2 phosphorylation, relief of inhibitory effect on mTORC1, subsequent higher mTORC1 activation and phosphorylation of its key substrate S6K1. (B) Compensatory mechanism following TRAF2 knockout that results in no change in mTORC1 activity dynamics as compared to WT cells: TRAF2 deletion reduces mTORC1 formation and abundance because of lower ubiquitinated MLST8 but at the same time leads to stronger mTORC1 activity (per molecule) due to higher AKT phosphorylation; and the net effect is no significant change in the phosphorylation of S6K1.
(TIF)

S4 Fig Dose-response simulations of the steady-state values of pS6K1 and pAKT S473 in response to increasing raptor, OTUD7B or MLST8. Total raptor, OTUD7B and MLST8 concentrations were perturbed within 100 folds up/down of their initial values. The pS6K1 and pAKT S473 curves for each parameter set were normalized by their corresponding maximal (peak) values and then average and S.E.M. were calculated at each concentration.
(TIF)

S5 Fig A reaction schematic of model 4 with overlay of the kinetic parameters. Normal arrows indicate positive regulation, bar-headed arrows indicate negative regulation. The red lines indicate the links that exert strongest impacts on the *biphasicness* (i.e. BI) of the SIN1-mTORC1 dependency.
(TIF)

S6 Fig Biphasic response gives rise to highly context-dependent effect of mTORC2 blockade. Depending on the initial state of mTORC2 (e.g. SIN1 level) and the efficiency of blockade, mTORC2 inhibition may result in upregulation, downregulation, or even no change in the phosphorylation level of the key mTORC1 substrate S6K1. This potentially explains the diverse, seemingly conflicting effect of mTORC2 blockade on mTORC1 activity reported in multiple previous studies.
(TIF)

S7 Fig Comparison of model performance of the original and modified model 4, which describe activation of mTORC2 by pAKT T308 or pIRS1/2, respectively. (A) Model simulations (blue curves) using best-fitted parameter sets as compared to the quantified experimental data (red curves). (B) Simulations of the dynamic response of various network components to insulin stimulation in control (WT MEF cells, blue lines) condition and when TRAF2 is deleted (TRAF2-/- MEF cells, red lines), in comparison to the corresponding experimental data (right panels), shown for the modified and original model 4.
(TIF)

S8 Fig Result of the 500 independent GA-based optimization runs for the four models. Per model, each GA run produced a best-fitted parameter set, which were then sorted by their corresponding objective function values. The threshold cut-off was determined based on visual assessment of fitting quality, and the parameter sets with objective function value under this threshold were selected for further analysis.
(TIF)

S9 Fig Clustering the best-fitted parameter sets of Model 4. Numbers on the right indicate objective function values corresponding to the parameter sets. OFVs: Objective Function Values.
(TIF)

S10 Fig Inferred mean concentration and standard errors of the model proteins based on analysing CCLE and iBAQ data for four representative cell lines. (TIF)

S1 Table Reactions and rate equations of the PI3K/MTOR pathway model. (DOCX)

S2 Table Ordinary differential equations of the PI3K/MTOR pathway model. The reaction rates are given in S1 Table. The initial conditions are representative values.
(DOCX)

S1 File Best fitted parameter sets for Models 1–4. (XLSX)
