# Supplementary figures and images for: Dynamic modelling of the PI3K/MTOR signalling network uncovers biphasic dependence of mTORC1 activity on the mTORC2 subunit SIN1
Source: PLoS Comput Biol. 2021 Sep 16;17(9):e1008513. doi: 10.1371/journal.pcbi.1008513 (PMC8478217; doi:10.1371/journal.pcbi.1008513)

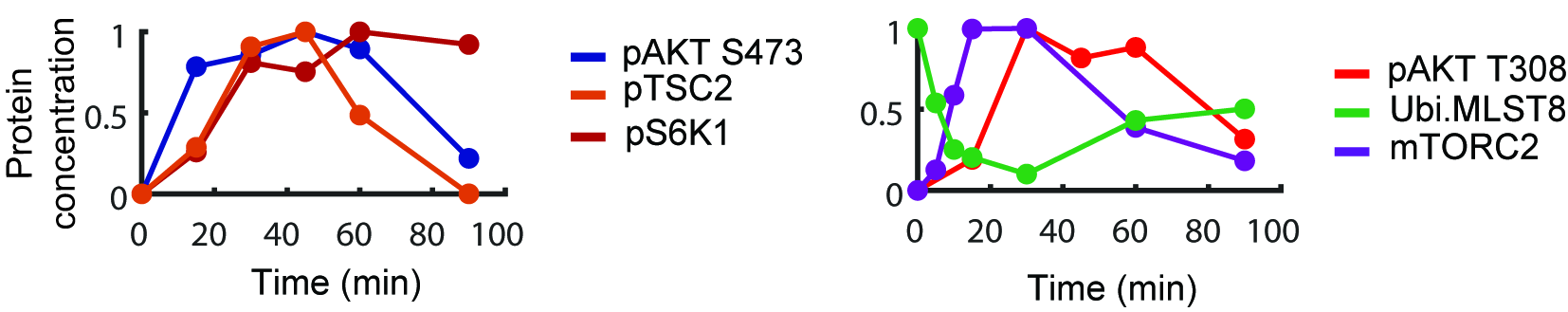

Supplement: S1 Fig — Phosphorylated (p) and ubiquitinated (Ubi) levels of each protein were normalized to the corresponding total protein levels. Each curve was normalized by their maximal (peak) value. (TIF) [file pcbi.1008513.s001.tif]

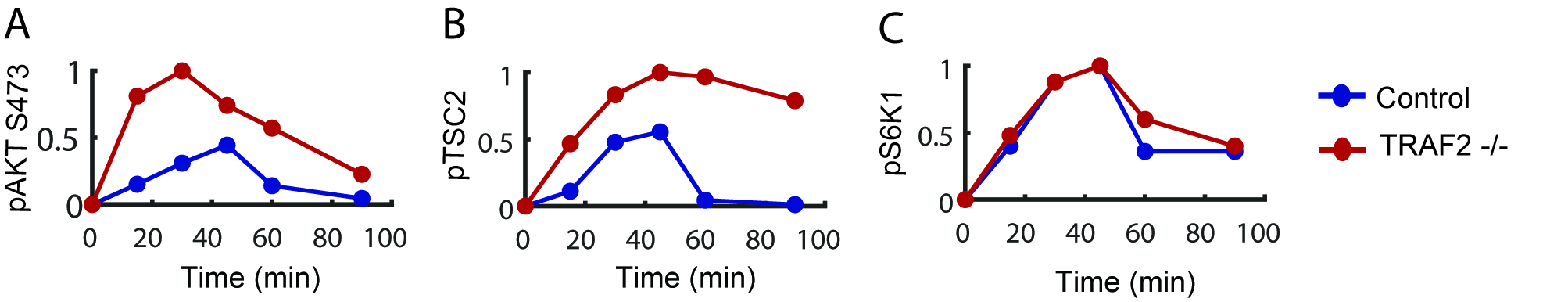

Supplement: S2 Fig — Phosphorylated (p) levels of each protein were normalized to the corresponding total protein levels. The curves under TRAF2 knockout condition were normalized by their corresponding maximal (peak) values, and the WT curves were scaled accordingly. (TIF) [file pcbi.1008513.s002.tif]

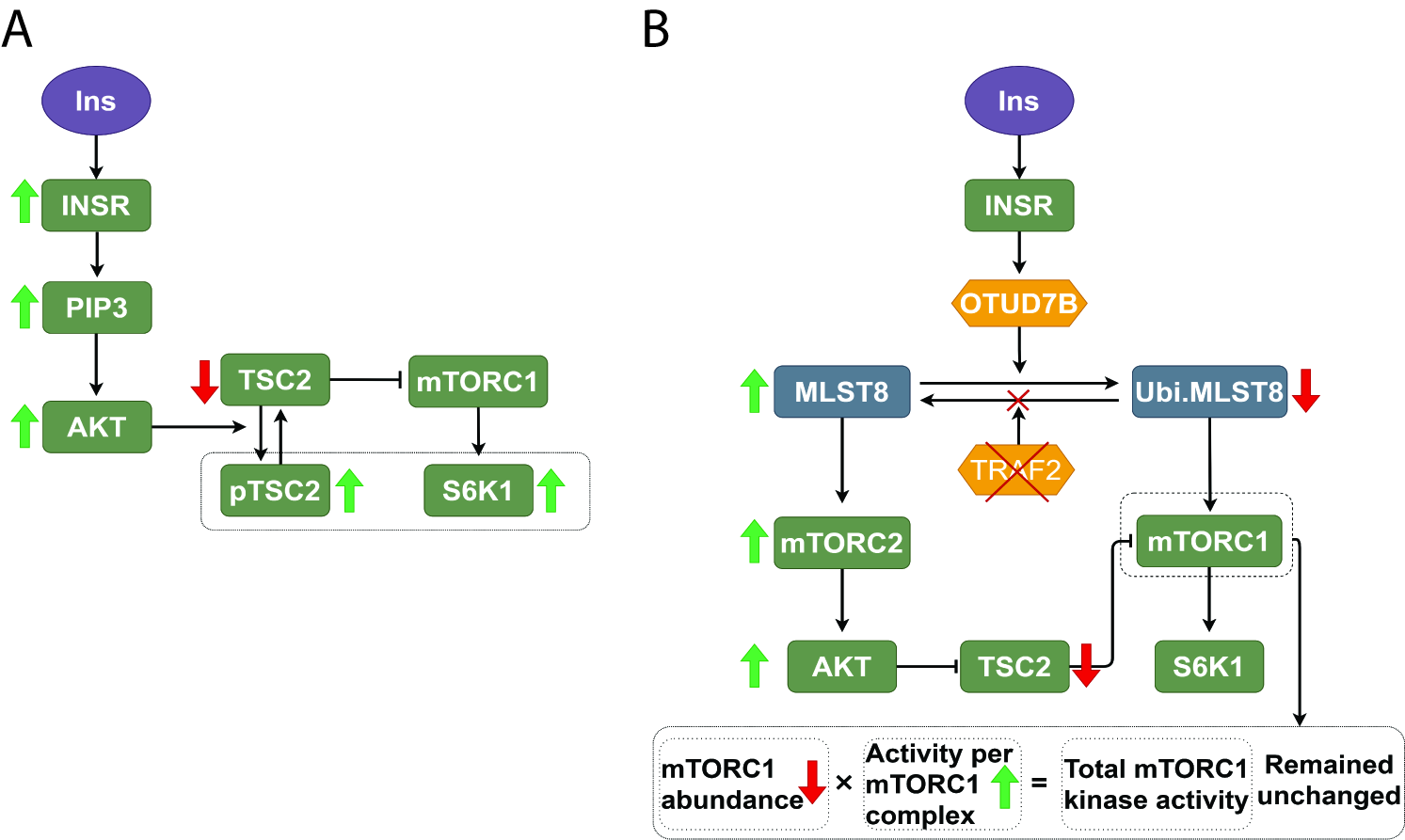

Supplement: S3 Fig — (A) In response to insulin stimulation, phosphorylated AKT increases. This leads to enhanced TSC2 phosphorylation, relief of inhibitory effect on mTORC1, subsequent higher mTORC1 activation and phosphorylation of its key substrate S6K1. (B) Compensatory mechanism following TRAF2 knockout that results in no change in mTORC1 activity dynamics as compared to WT cells: TRAF2 deletion reduces mTORC1 formation and abundance because of lower ubiquitinated MLST8 but at the same time leads to stronger mTORC1 activity (per molecule) due to higher AKT phosphorylation; and the net effect is no significant change in the phosphorylation of S6K1. (TIF) [file pcbi.1008513.s003.tif]

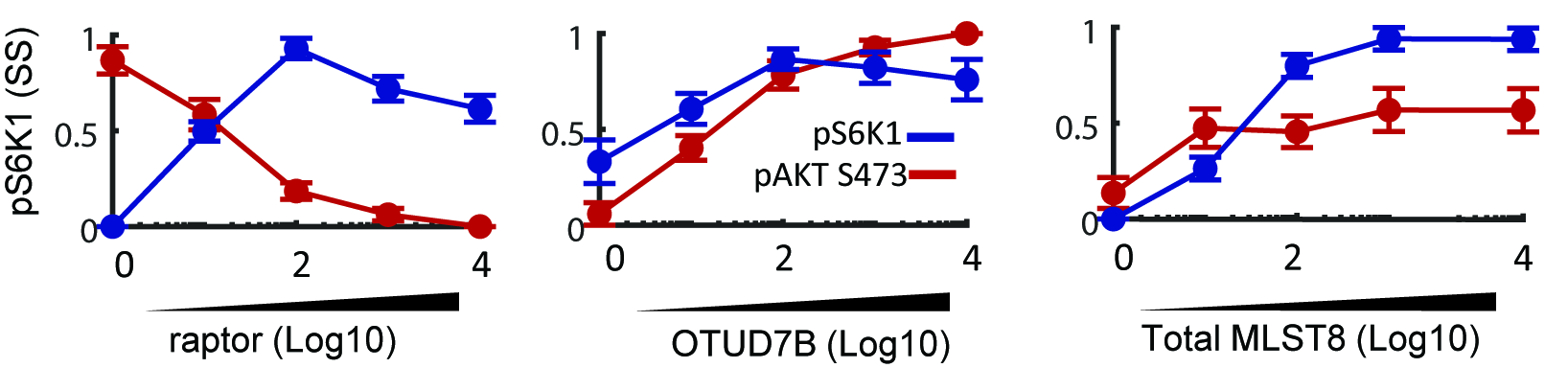

Supplement: S4 Fig — Total raptor, OTUD7B and MLST8 concentrations were perturbed within 100 folds up/down of their initial values. The pS6K1 and pAKT S473 curves for each parameter set were normalized by their corresponding maximal (peak) values and then average and S.E.M. were calculated at each concentration. (TIF) [file pcbi.1008513.s004.tif]

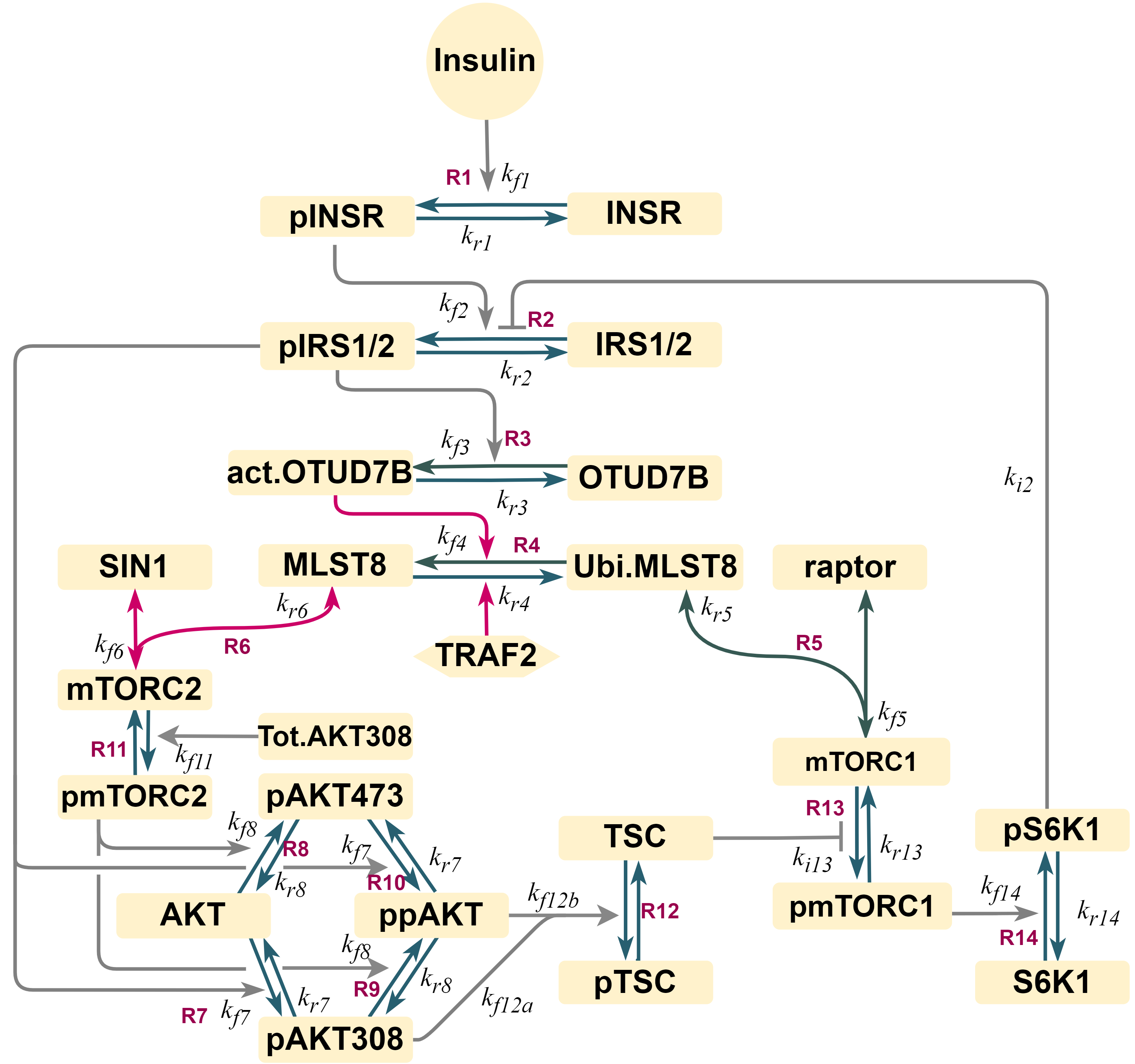

Supplement: S5 Fig — Normal arrows indicate positive regulation, bar-headed arrows indicate negative regulation. The red lines indicate the links that exert strongest impacts on the biphasicness (i.e. BI) of the SIN1-mTORC1 dependency. (TIF) [file pcbi.1008513.s005.tif]

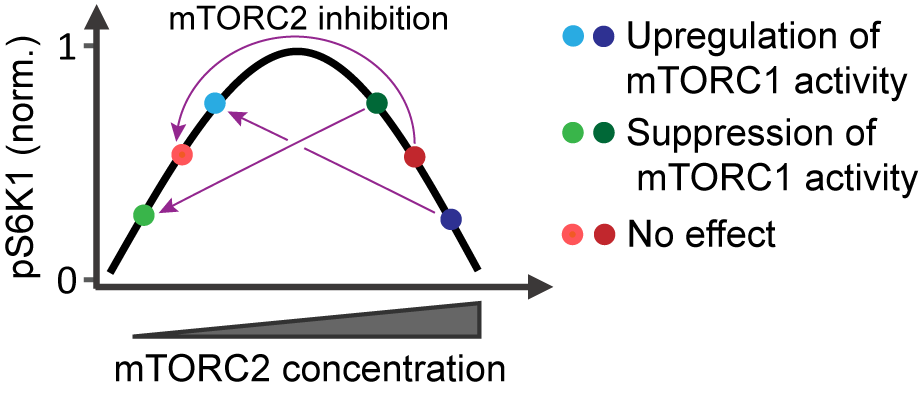

Supplement: S6 Fig — Depending on the initial state of mTORC2 (e.g. SIN1 level) and the efficiency of blockade, mTORC2 inhibition may result in upregulation, downregulation, or even no change in the phosphorylation level of the key mTORC1 substrate S6K1. This potentially explains the diverse, seemingly conflicting effect of mTORC2 blockade on mTORC1 activity reported in multiple previous studies. (TIF) [file pcbi.1008513.s006.tif]

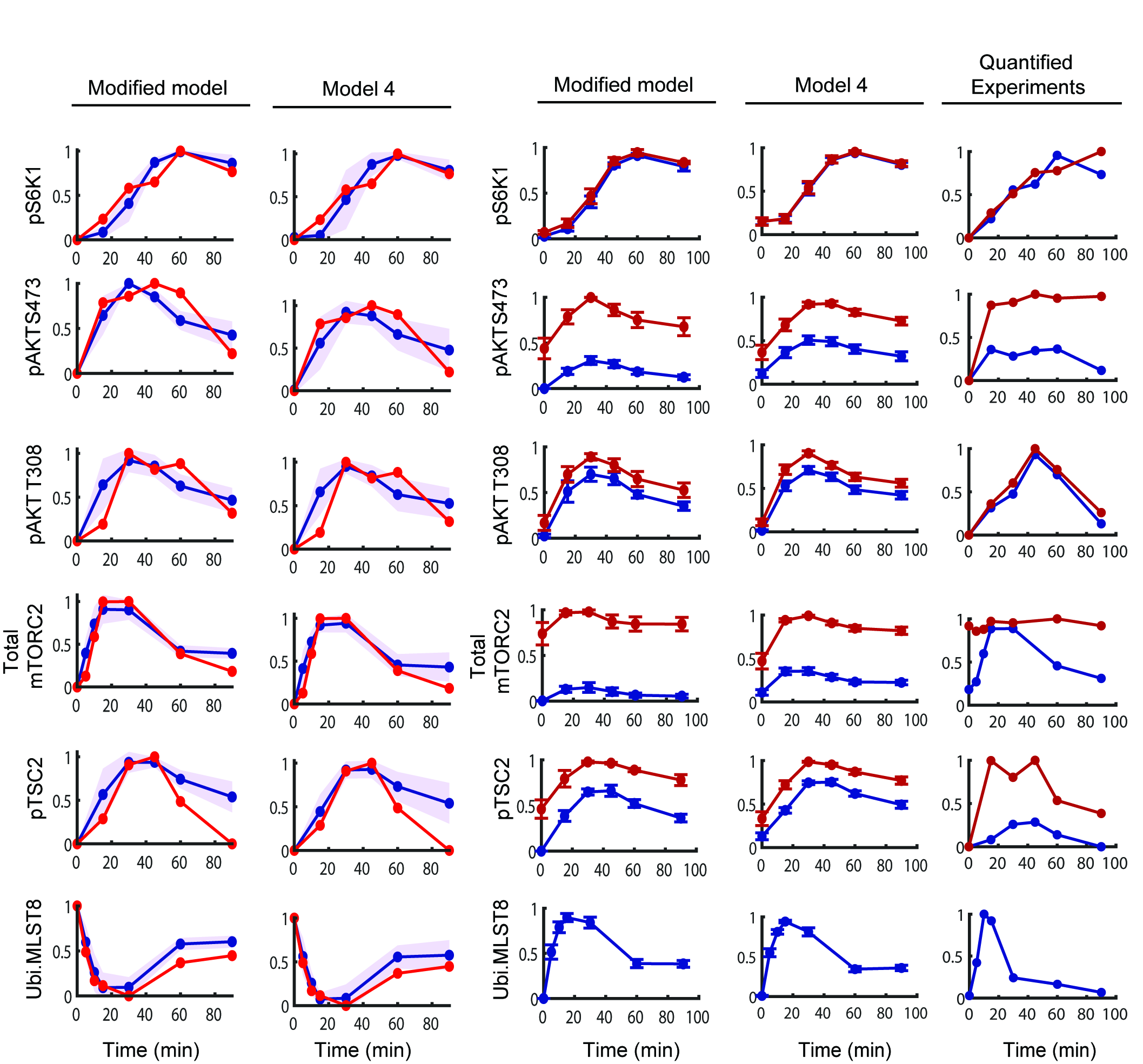

Supplement: S7 Fig — (A) Model simulations (blue curves) using best-fitted parameter sets as compared to the quantified experimental data (red curves). (B) Simulations of the dynamic response of various network components to insulin stimulation in control (WT MEF cells, blue lines) condition and when TRAF2 is deleted (TRAF2-/- MEF cells, red lines), in comparison to the corresponding experimental data (right panels), shown for the modified and original model 4. (TIF) [file pcbi.1008513.s007.tif]

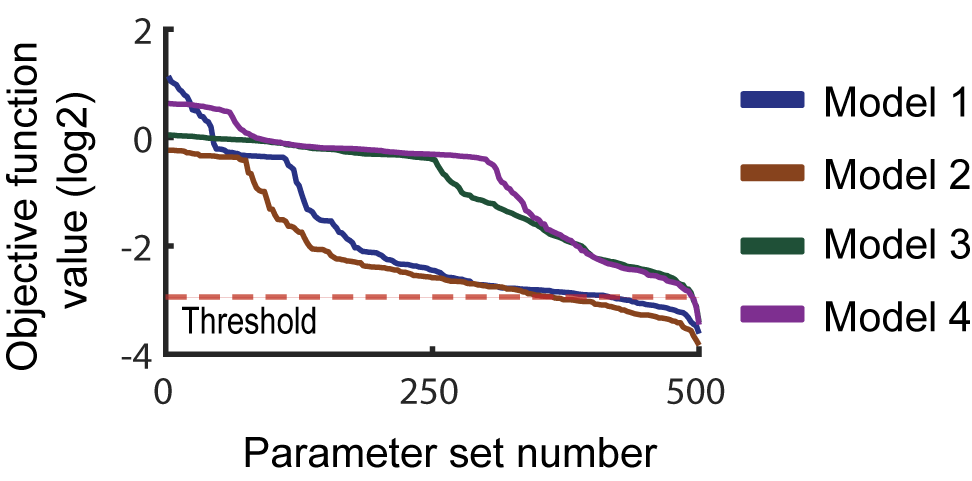

Supplement: S8 Fig — Per model, each GA run produced a best-fitted parameter set, which were then sorted by their corresponding objective function values. The threshold cut-off was determined based on visual assessment of fitting quality, and the parameter sets with objective function value under this threshold were selected for further analysis. (TIF) [file pcbi.1008513.s008.tif]

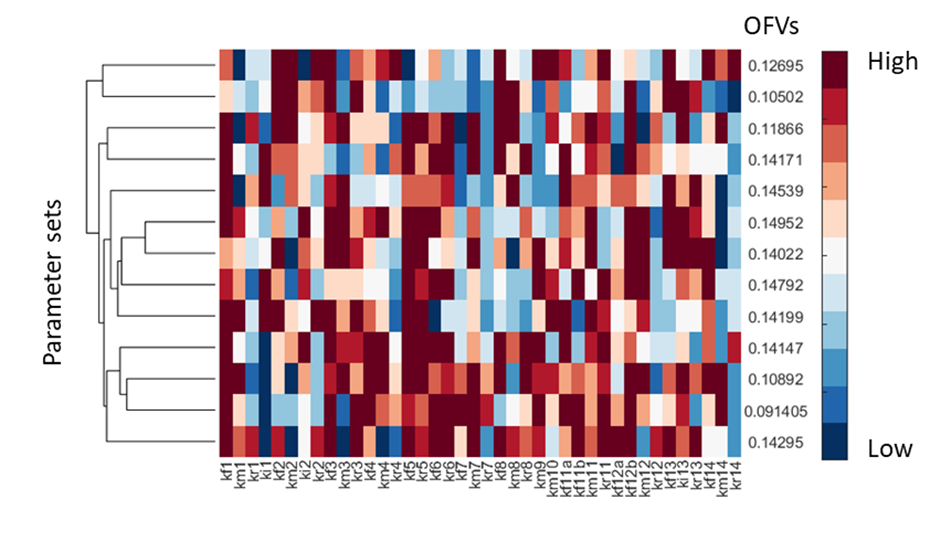

Supplement: S9 Fig — Numbers on the right indicate objective function values corresponding to the parameter sets. OFVs: Objective Function Values. (TIF) [file pcbi.1008513.s009.tif]

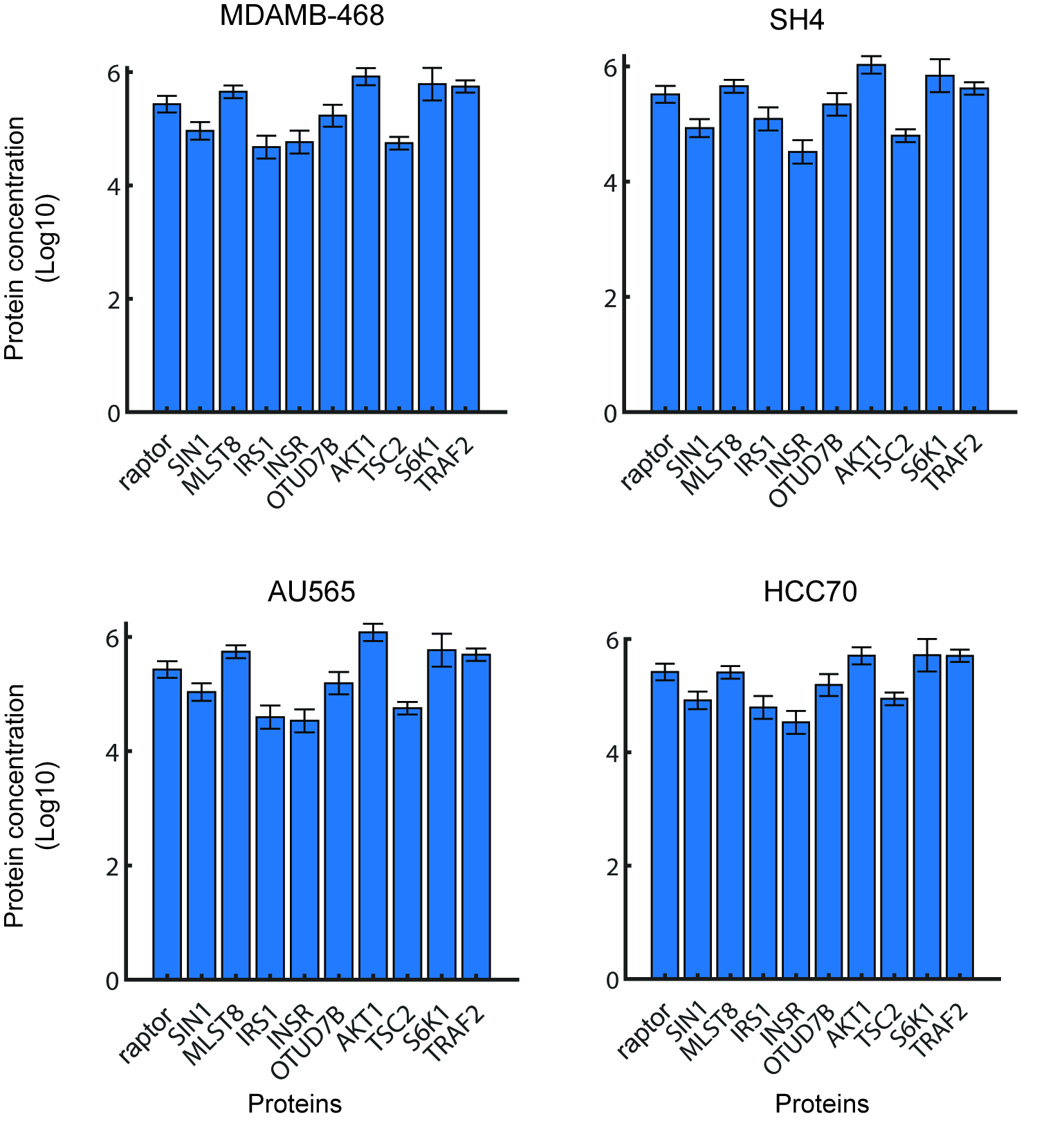

Supplement: S10 Fig — (TIF) [file pcbi.1008513.s010.tif]
