# Supplementary material for: Dynamic modelling of the PI3K/MTOR signalling network uncovers biphasic dependence of mTORC1 activity on the mTORC2 subunit SIN1
Source: PLoS Comput Biol. 2021 Sep 16;17(9):e1008513. doi: 10.1371/journal.pcbi.1008513 (PMC8478217; doi:10.1371/journal.pcbi.1008513)
Supplement: S1 Table — (DOCX) [file pcbi.1008513.s011.docx]

**SUPPLEMENTARY TABLES**

**S1 Table. Reactions and rate equations of the PI3K/AKT pathway model**

|  | **Reactions** | **Reaction rates** |
| --- | --- | --- |
| R1 | InsR ↔ pInsR | kf1 * InsR*(Ins+InsB0)/(Km1 + InsR) - kr1*pInsR |
| R2 | IRS ↔ pIRS | kf2*IRS*pInsR/((Km2 + IRS)*(1+ ki2*pS6K1)) - kr2*pIRS |
| R4 | Ubi.mLST8 ↔ mLST8 | kf4 * act.OTUD7B * ubi.mLST8/(km4+ ubi.mLST8) - kr4* mLST8 *TRAF |
| R6 | mLST8 + Sin1 ↔ mTORC2 | Kf6* mLST8 * Sin1 – kr6*mTORC2 |
| R7 | Akt ↔ pAkt308 | kf7 * pIRS * Akt / (Km7 + Akt) - kr7 * pAkt308 |
| R8 | Akt ↔ pAkt473 | Kf8 * pmTORC2 *Akt/ (Km8 + Akt) – kr8 * pAkt473 |
| R9 | pAkt308 ↔ pAkt308473 | Kf8 * pmTORC2* pAkt308 / (Km9 + pAkt308) – kr8 * pAkt308473 |
| R10 | pAkt473 ↔ pAkt308473 | Kf7 * pIRS * pAkt473 / (Km10 + pAkt473) – kr7 * pAkt308473 |
| R11 | mTORC2 ↔ pmTORC2 | (kf11a* pAkt308 + kf11b* pAkt308473 ) * mTORC2/ (Km11 + mTORC2) - kr11 * pmTORC2 |
| R12 | TSC2 ↔ pTSC2 | TSC2*(kf12a*pAkt308 + kf12b*pmAkt308473)/(Km12 + TSC2)-kr12*pTSC2 |
| R13 | mTORC1 ↔ pmTORC1 | kf13 * mTORC1/(1+ki13*TSC2) - kr13*pmTORC1 |
| R14 | S6K1 ↔ pS6K1 | kf14 * mTORC1* S6K1 / (Km14 + S6K1) - kr14* pS6K1 |

| **Models** |  | **Reactions** | **Reaction rates** |
| --- | --- | --- | --- |
| Model 1 | R3 | OTUD7B ↔ act.OTUD7B | kf3 * pInsR * OTUD7B /(km3+ OTUD7B) - kr3*act.OTUD7B |
| Model 2 | R3 | OTUD7B ↔ act.OTUD7B | kf3 * pInsR * OTUD7B /(km3+ OTUD7B) - kr3*act.OTUD7B |
| Model 3 | R3 | OTUD7B ↔ act.OTUD7B | kf3 * pInsR * OTUD7B /(km3+ OTUD7B) - kr3*act.OTUD7B |
|  | R5 | Ubi.mLST8 + Raptor ↔ mTORC1 | kf5* Ubi.mLST8 *Raptor - kr5*mTORC1 |
| Model 4 | R3 | OTUD7B ↔ act.OTUD7B | kf3 * pInsR * OTUD7B /(km3+ OTUD7B) - kr3*act.OTUD7B |
|  | R5 | Ubi.mLST8 + Raptor ↔ mTORC1 | kf5* Ubi.mLST8 *Raptor - kr5*mTORC1 |
