# Supplementary material for: Dynamic modelling of the PI3K/MTOR signalling network uncovers biphasic dependence of mTORC1 activity on the mTORC2 subunit SIN1
Source: PLoS Comput Biol. 2021 Sep 16;17(9):e1008513. doi: 10.1371/journal.pcbi.1008513 (PMC8478217; doi:10.1371/journal.pcbi.1008513)
Supplement: S2 Table — The reaction rates are given in S1 Table. The initial conditions are representative values. (DOCX) [file pcbi.1008513.s012.docx]

**S2 Table. Ordinary differential equations of the PI3K/Akt pathway model. The reaction rates are given in S1 Table. The initial conditions are representative values.**

| **Left hand Sides** | **Right hand Sides** | **Left hand Sides** | **Right hand Sides** |
| --- | --- | --- | --- |
| d[InsR]/dt | -R1 | d[mTORC1]/dt | +R5-R13 |
| d[pInsR]/dt | +R1 | d[pmTORC1]/dt | +R13 |
| d[IRS]/dt | -R2 | d[Akt]/dt | -R7-R8 |
| d[pIRS]/dt | +R2 | d[pAkt473]/dt | +R8-R10 |
| d[OTUD7B]/dt | -R3 | d[pAkt308]/dt | +R7-R9 |
| d[act.OTUD7B]/dt | +R3 | d[pAkt308473]/dt | R9+R10 |
| d[mLST8]/dt | R4 - R6 | d[S6K1]/dt | -R14 |
| d[Ubi.mLST8]/dt | -R4-R5 | d[pS6K1]/dt | R14 |
| d[Raptor]/dt | -R5 | d[TSC2]/dt | -R12 |
| d[Sin1]/dt | -R6 | d[pTSC2]/dt | R12 |
| d[mTORC2]/dt | -R11 |  |  |
| d[pmTORC2]/dt | R11 |  |  |
